# Recent Strategies in Transition-Metal-Catalyzed Sequential C–H Activation/Annulation for One-Step Construction of Functionalized Indazole Derivatives

**DOI:** 10.3390/molecules27154942

**Published:** 2022-08-03

**Authors:** Pezhman Shiri, Atefeh Roosta, Wim Dehaen, Ali Mohammad Amani

**Affiliations:** 1 Department of Medical Nanotechnology, School of Advanced Medical Sciences and Technologies, Shiraz University of Medical Sciences, Shiraz 7133654361, Iran; amani_a@sums.ac.ir; 2 Pharmaceutical Sciences Research Center, Shiraz University of Medical Sciences, Shiraz 7133654361, Iran; 3 Department of Chemistry, Tarbiat Modares University, Tehran 1411713116, Iran; atefehroosta862@gmail.com; 4 Molecular Design and Synthesis, Department of Chemistry, KU Leuven, 3001 Leuven, Belgium

**Keywords:** C–H functionalization, cyclization, indazole products, transition metals catalysis

## Abstract

Designing new synthetic strategies for indazoles is a prominent topic in contemporary research. The transition-metal-catalyzed C–H activation/annulation sequence has arisen as a favorable tool to construct functionalized indazole derivatives with improved tolerance in medicinal applications, functional flexibility, and structural complexity. In the current review article, we aim to outline and summarize the most common synthetic protocols to use in the synthesis of target indazoles via a transition-metal-catalyzed C–H activation/annulation sequence for the one-step synthesis of functionalized indazole derivatives. We categorized the text according to the metal salts used in the reactions. Some metal salts were used as catalysts, and others may have been used as oxidants and/or for the activation of precatalysts. The roles of some metal salts in the corresponding reaction mechanisms have not been identified. It can be expected that the current synopsis will provide accessible practical guidance to colleagues interested in the subject.

## 1. Introduction

**Figure 1:**
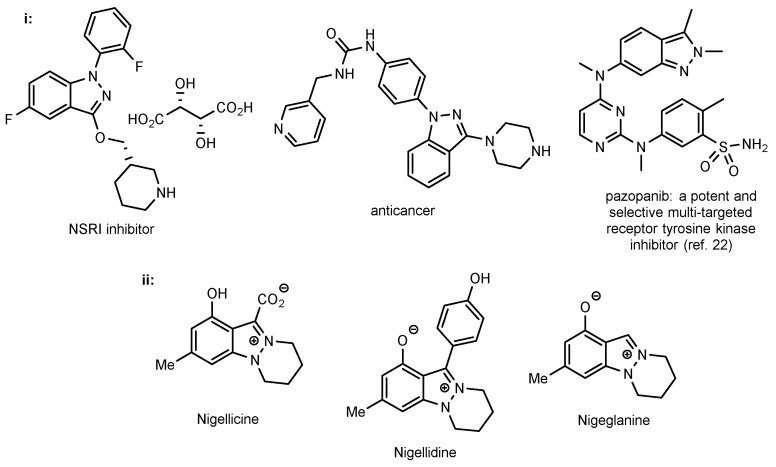
(**i**) Indazole derivatives with biological activity; (**ii**) Natural products of indazole alkaloids.

Nitrogen-based heterocyclic systems are commonly found in pharmaceutical agents and natural products, and they have been intensively explored as new bioactive products [1,2,3,4,5,6,7,8,9,10]. Among them, the indazoles [11,12,13,14,15,16] are favored by synthetic and medicinal chemists, as evidenced by their widespread abundance in pharmaceuticals and natural products. In fact, products bearing the indazole segment have been found to show diverse biological activities, including analgesic, anticancer, anesthetic, anti-inflammatory, antifungal, anti-HIV, antiarrhythmic, and anti-emetic properties and high binding affinity for estrogen receptors (Figure 1) [11,17,18,19,20,21,22,23,24]. Pazopanib with an indazole scaffold has been proven to be a potent and selective multitargeted receptor tyrosine kinase inhibitor that inhibits tumor growth [25]. Moreover, derivatives of indazole have been used as electronically active and agricultural materials [26,27]. Indazoles are found in nature as well, and the structures of three natural products containing the indazole scaffold, named nigellicine, nigellidine, and nigeglanine, are illustrated in Figure 1 [11,28,29,30,31].

Notably, the known synthetic procedures toward indazoles may suffer from multistep routes, severe conditions, and relatively low substrate diversity [32,33,34]. Therefore, considerable attempts have been devoted to constructing these biologically critical scaffolds more efficiently, although the exploration of novel atom-economical strategies to produce these unique scaffolds is still a challenging topic. Recently, a transition-metal-catalyzed C−H bond activation and cyclization sequence [35,36,37,38,39,40] has received significant attention to provide these structures.

Transition-metal-catalyzed C−H activation [41] and annulation sequences [37,42,43,44,45,46,47,48] are powerful and reliable tools for the formation of complex molecular structures in an efficient, economical, practicable, and straightforward manner [37,49]. These synthetic strategies provide highly atom- and step-economic transformations for the development of efficient synthetic procedures with high functional-group tolerance while avoiding the excessive prefunctionalization of reactive centers. One major issue for these protocols is the intrinsic inferior reactivity of C−H bonds, requiring harsh conditions to convert substrates to the target products in satisfactory yields and resulting in low selectivities [50]. To solve this, the C−H activation of inactive arenes via a pendent chelating activator is an efficient route that is additionally incurring site selectivity in C−H activation. Metal salts or metal complexes have commonly been applied for these transformations. Thus, transition-metal-catalyzed sequential C–H activation/annulation reactions of suitable substrates with a variety of coupling partners have been applied to assemble complex indazole-based architectures.

In 2018, a general review appeared on recent advances in various methods for the synthesis of indazole derivatives, focusing on their biological activities [12]. Meanwhile, Li et al. outlined the anticancer activity of indazole derivatives [51] while also summarizing the synthetic protocols and structure–activity relationships of target products [51]. In 2021, Babu et al. comprehensively covered the recent developments in the transition-metal-catalyzed synthesis of indazoles [52]. An overview of the development of novel and green electrochemical approaches in the functionalization of indazole derivatives was reported by Hajra in 2021 [53]. Moreover, a review article for approved marketed drugs containing indazole scaffolds as valid preclinical/clinical drug compounds was published by Wu et al. in 2021 [54]. Because of the significance of indazole heterocyclic systems as well as the rapid development of strategies based on transition-metal-catalyzed sequential C–H activation/annulation for the one-step synthesis of functionalized indazole derivatives, a dedicated comprehensive overview would be timely and beneficial for future drug discovery.

Consequently, we aim to present a review arranged according to the various sorts of metal salts or metal complexes applied in such transition-metal-catalyzed C–H activation and annulation sequences. The purpose of the current overview is to report the recent exploration in this area based on different transition metal catalysts derived from rhodium, cobalt, palladium, rhenium, and copper. While covering the subject, a variety of examples and selected mechanisms of reactions are discussed.

## 2. Different Synthesis Routes for the Construction of Indazoles via Transition-Metal-Catalyzed Sequential C–H Activation/Annulation

### 2.1. Synthesis of Indazoles Using Rhodium and Copper Salts

**Scheme 1:**
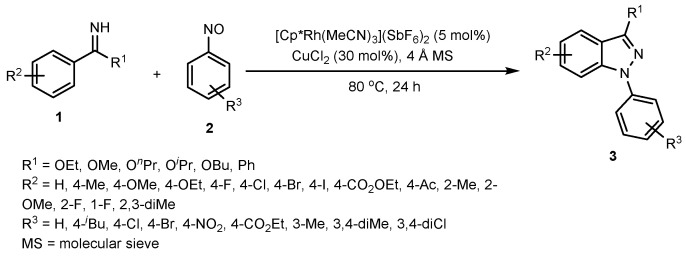
A synthetic route for the preparation of 1*H*-indazole derivatives **3** from the reaction between imidates **1** and nitrosobenzenes **2** in the presence of a rhodium/copper catalyst.

In 2016, a facile and efficient access to 1*H*-indazoles **3** was established through Rh(III)/Cu(II)-catalyzed sequential C–H bond activation and intramolecular cascade annulation. The reaction occurs at 80 °C within 24 h in PhCF_3_ as a solvent. A comprehensive examination of this process was conducted using ethyl benzimidates **1** and nitrosobenzenes **2**. A control experiment without adding Rh or Cu catalysts was then run to demonstrate that this transformation could not proceed without either one of these catalysts. The authors proposed a significant facilitation role of the bridging acetate ligand in the Rh_2_(OAc)_4_ structure for the C–H activation. Benzimidate scaffolds **1** with both electron-withdrawing and electron-donating functional groups as well as halogens all worked well to afford the desired products with moderate to high yields. Furthermore, imidate substrates **1** that bear other alkyl esters as well as aryl substitution were transformed into the corresponding products **3** smoothly. Subsequently, a diverse range of nitrosobenzenes **2** with electron-donating and -withdrawing groups at different positions of the aryl ring were also proven to be viable substrates. Significantly, this transformation features satisfactory functional-group tolerance with good to high yields (Scheme 1) [55].

**Scheme 2:**
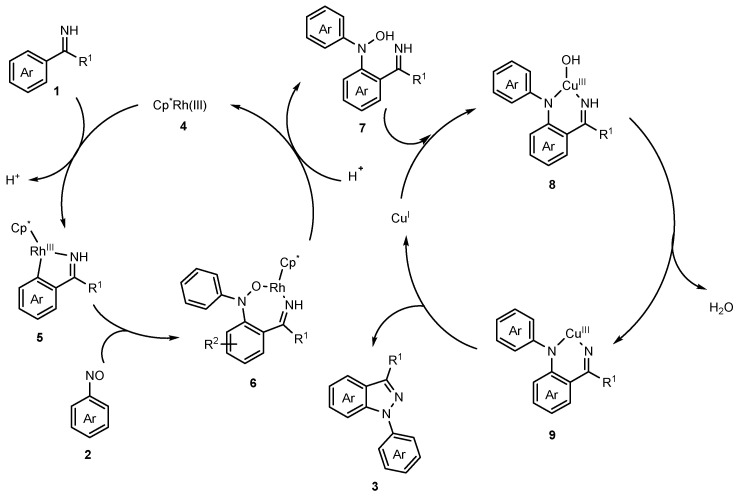
A rational mechanism for the synthesis of 1*H*-indazole derivatives **3** from the reaction between imidates **1** and nitrosobenzenes **2** in the presence of a rhodium/copper catalyst.

The mechanism for the formation of 1*H*-indazoles **3** through the reaction of imidates **1** with nitrosobenzenes **2** catalyzed by a rhodium/copper catalyst is given in Scheme 2. A rhodacycle **5** is generated by the coordination of an imidate **1** to a catalyst **4** and C−H activation. Subsequently, the migratory insertion of the Rh−C bond into the N=O group yields a six-membered rhodacycle **6**. In the next step, the protonolysis of the intermediate **6** delivers a hydroxylamine **7** along with a regenerated Rh(III) catalyst **4**. In another cycle, N−O oxidative addition is carried out using the Cu(I) catalyst to deliver a Cu(III) organocupracycle **8**. Finally, the dehydration of **8** and the reductive elimination of **9** take place to form the N−N bond [55]. Although the authors reported intermediates **8** and **9**, it seems Cu(III) would involve a chloride or another negatively charged ligand attached to copper in intermediates **8** and **9**.

**Scheme 3:**
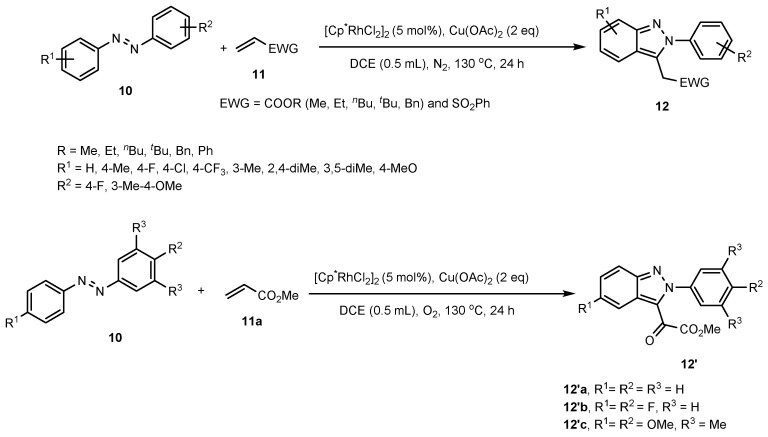
A synthetic route for the transformation of azobenzene substrates **10** to indazole derivatives **11** in the presence of a Rh(III) catalyst.

A Rh(III)-mediated substrate-controlled conversion of azobenzenes **10** and alkenes **11** to indazoles **12** was described via C–H functionalization and cyclization (Scheme 3). First, 5 mol% of [Cp*RhCl_2_]_2_ and 200 mol% of Cu(OAc)_2_ as oxidant and DCE as solvent were utilized to transfer azobenzenes **10** to indazoles **12** under nitrogen atmosphere in good to excellent yields. To highlight the importance of having both metals present, no desired compound was formed when either the Rh(III) as catalyst or Cu(II) as oxidant were removed. The scope of this C–H functionalization/cyclization reaction with regard to both azobenzenes **10** and alkenes **11** was screened. Acrylates **11** with different substitutions efficiently proceeded to afford final products in satisfactory yields. However, phenyl vinyl sulfone and dimethyl vinyl phosphonate did not react with its azobenzene partner under optimized conditions. Azobenzene substrates possessing both electron-donating and electron-withdrawing functional groups were well-tolerated, as evidenced by the isolation of the desired products in moderate to good yields. The product with a stronger electron-withdrawing substituent (CF_3_) was formed only in a trace amount. Next, this coupling reaction was applied to *m*- or *o*-substituted azobenzenes. The *m*-Me azobenzene **10** regioselectively resulted in the generation of the corresponding product **12** after reacting at the less hindered position. However, the *m*-MeO azobenzene **10** led to a mixture of two regioisomers **12**. The treatment of symmetrical disubstituted azobenzenes **10** with alkenes **11** enabled the C–H functionalization/cyclization sequence in good to moderate yields, while unsymmetrically substituted azobenzene **10** afforded the corresponding product **12**, after reaction at the less hindered position, in a good yield. Surprisingly, 3-acyl indazole products **12′** were achieved by performing the reactions under oxygen atmosphere. The subsequent utilization of the present strategy could be a rapid and straightforward approach for the synthesis of new functional and biologically active molecules [56].

**Scheme 4:**
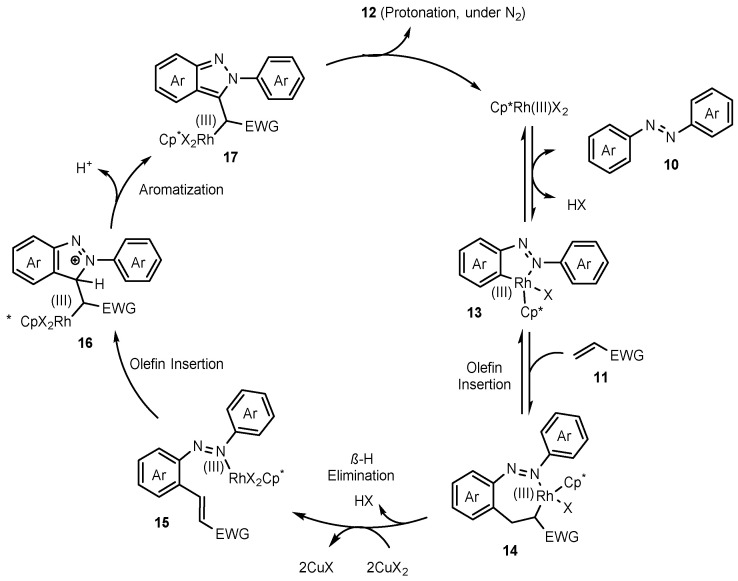
A possible mechanism for the transformation of azobenzene substrates **10** to indazole derivatives **12** in the presence of a Rh(III) catalyst.

The mechanism for the synthesis of indazoles **12** from azobenzenes **10** is exhibited in Scheme 4, which involves coordination, C−H activation, alkene coordination and insertion, *β*-hydride elimination, the insertion of a C=C bond into the Rh-N bond, and then aromatization to produce indazoles. It was proposed that copper acetate plays its role in the step of the *β*-hydride elimination to give indazoles [56].

**Scheme 5:**
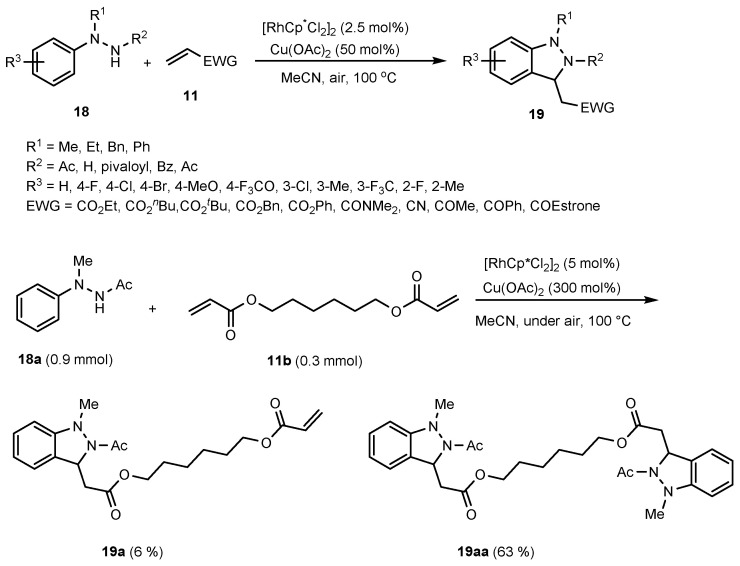
A synthetic route for the preparation of 2,3-dihydro-1*H*-indazole derivatives **19** from the reaction between 1,2-disubstituted arylhydrazines **18** and electron-poor alkenes **11** in the presence of a Rh(III) catalyst.

A novel methodology for the synthesis of 2,3-dihydro-1*H*-indazoles **19** via the oxidative olefination of 1,2-disubstituted arylhydrazines **18** with alkenes **11** through C(sp^2^)–H bond functionalization pursued by an intramolecular aza-Michael transformation was reported by Kim et al. A series of highly substituted 2,3-dihydro-1*H*-indazoles **19** with different substituents were obtained in low to high yields in the presence of [RhCp*Cl_2_]_2_ as a catalyst and Cu(OAc)_2_ as an oxidant in MeCN under air atmosphere at 100 °C for 20 h in pressure tubes. The substrate scope was subsequently explored under this catalytic system, as illustrated in Scheme 5. Various acrylates **11** and *N*′-methyl-*N*′-arylacetohydrazides **18** with both electron-donating and -withdrawing substituents at the para-site of the aromatic ring were explored, giving the desired products **19** via oxidative olefination and the subsequent intramolecular cyclization in moderate to good yields. The functional-group tolerance, especially to the bromo and chloro groups, provides a versatile synthetic protocol for the further functionalization of the 2,3-dihydro-1*H*-indazoles **19**. Interestingly, when meta-substituted arylacetohydrazides **19** were used as the reaction substrates, Rh(III)-catalyzed oxidative coupling preferentially occurred at the less hindered site, providing the corresponding products **19** in a regioselective manner. Moreover, ortho-substituted compounds **18** afforded the desired products with slightly decreased effectivity, which is presumably attributed to the steric effect of a substituent at the ortho-position. The corresponding products **19** were further produced in low to high yields by the utilization of diverse olefins **11**. For acrylates containing -CO_2_Me, -CO_2_nBu, -CO_2_*^t^*Bu, -Bn, and -CO_2_Ph as EWGs and acrylamide (containing -CONMe_2_ as an EWG), 2,3-dihydro-1*H*-indazoles **19** were generated in high yields, while acrylonitrile **11** (containing -CN as an EWG) and but-3-en-2-one **11** (containing -COMe as an EWG) were reacted with *N*′-methyl-*N*′-phenylacetohydrazide with significantly decreased yield under the standard conditions. In the case of acrylate containing an estrogen scaffold, the desired product **19** was obtained in the reaction with *N*′-methyl-*N*′-phenylacetohydrazide. The bis-indazole **19aa** was then achieved in 63% yield by increasing the amounts of hydrazide, rhodium as a catalyst, and copper as an oxidant [57].

**Scheme 6:**
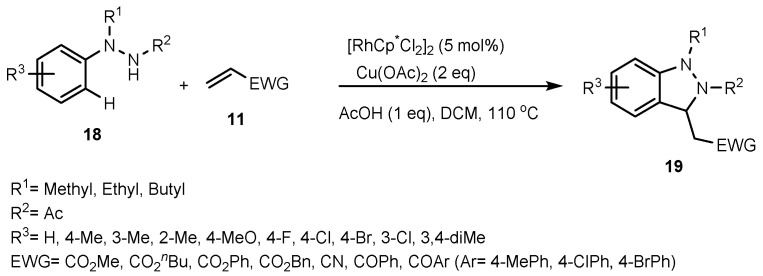
A synthetic route for the preparation of 2,3-dihydro-1*H*-indazoles **19** via the C–H cleavage of arylhydrazines **18** in the presence of a Rh(III) catalyst.

The C–H bond activation of *N*′-alkyl-*N*′-arylacetohydrazide **18,** followed by cyclization with a variety of acrylates, ethyl acrylate, methyl acrylate, *n*-butyl acrylate, *t*-butyl acrylate, phenyl acrylate, and benzyl acrylate **11,** has been performed for the preparation of 2,3-dihydro-1*H*-indazoles **19** using a catalytic amount of [Cp*RhCl_2_]_2_ in the presence of Cu(OAc)_2_ and AcOH in DCE at 110 °C within 24 h under N_2_ atmosphere. As illustrated in Scheme 6, the structurally diverse alkenes **19** reacted with *N*′-methyl-*N*′-phenylacetohydrazide to afford indazole derivatives **19** in good to high yields. A series of the *N*′-alkyl-*N*′-arylacetohydrazide derivatives **18** can smoothly be converted into corresponding products **19** with moderate to good yields as well. Disubstituted *N*′-alkyl-*N*′-arylacetohydrazides and *N*′-alkyl-*N*′-arylacetohydrazides **18** bearing both electron-rich and electron-deficient groups including methyl, methoxy, fluoro, chloro, and bromo on the aryl ring all displayed moderate to good reactivities in the sequence smoothly furnishing the desired indazoles **19**. In the case of meta-substituted *N*′-alkyl-*N*′-arylacetohydrazides, the reaction proceeded successfully at the less steric side of the arenes. Moreover, **18** containing an ortho-substituent also participated in this reaction to afford the corresponding product, although the yield greatly decreased. By having ethyl and *n*-butyl instead of methyl at the *N*-atom, the corresponding products **19** were obtained in moderate yields. This transformation provided a practical procedure to achieve useful target products **19** through C–H bond activation, featuring good to excellent yields with excellent regioselectivity, high atom economy, and versatile derivatization [58].

**Scheme 7:**
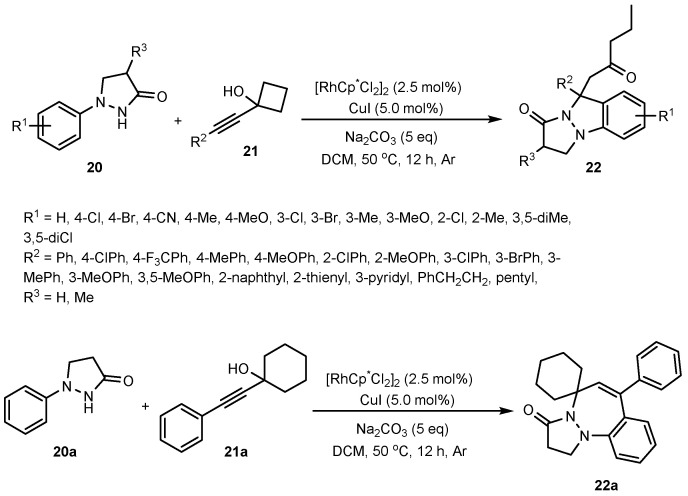
A synthetic route for the preparation of pyrazolo[1,2-*a*] indazoles **22** containing a quaternary carbon in the presence of a Rh(III) catalyst.

A catalytic reaction of 1-alkynylcyclobutanols **20** with pyrazolidinones **21** toward pyrazolo [1,2-*a*] indazoles **22** bearing a quaternary carbon was developed by Yu et al. in 2020. The reaction proceeds through Rh(III)-catalyzed sequential C−H/N-H activation and ring opening, followed by cyclization transformation in a one-pot process. This protocol provides an efficient and atom-economical approach for the direct synthesis of pyrazolo[1,2-*a*] indazoles **22** in high yields with exclusive regioselectivity under the optimized conditions (using catalytic amounts of [Cp*RhCl_2_]_2_ and CuI in the presence of Na_2_CO_3_ in DCE at 50 °C within 12 h under Ar atmosphere) (Scheme 7). First, the reaction between 1-phenylpyrazolidin-3-one **20** and 1-alkynylcyclobutanols **21** bearing several electron-rich and electron-poor substituents at different positions on the phenyl ring was explored, affording the desired products in good to high yields. When the standard reaction conditions were applied to 1-alkynylcyclobutanols **21** with para- and meta-substituted phenyl groups, high yields of products were obtained. The usage of 1-(phenylethynyl) cyclobutanols **20** containing an electron-poor or electron-rich group, including halide or methoxy at the ortho-position of the benzene had little effect, and pyrazolo[1,2-*a*] indazole derivatives were achieved with good yields. Even the substrates substituted with heterocyclic naphthyl, thienyl, and pyridyl groups or alkyl groups could be used in the coupling reaction with satisfactory yields. Surprisingly, by the replacement of the cyclobutanol moiety of 1-(phenylethynyl) cyclobutanol with cyclohexanol, a seven-membered ring product **22a** was obtained with good yield. In order to better understand the substrate scope of this [4 + 1] cyclization and ring opening, a series of *N*-arylpyrazolidinones **20** were further tested under standard conditions. The coupling transformations proceeded successfully to form the desired indazoles **22** in moderate and high yields by the introduction of substituents such as -Cl, -Br, -CN, -Me, or -OMe at the para-site and -Cl, -Br, -Me, -OMe at the meta-site of the aryl ring. The *m*-OMe-substituted pyrazolidinone **20** displayed slightly lower regioselectivity for this reaction. Two pyrazolidinones substituted at the 3- and 5-positions were examined, and these delivered the desired products in reasonable yields. However, the [4 + 1] annulation and ring opening failed to form the corresponding products **22** when the ortho-substituted pyrazolidinones **20** possessing steric hindrance were exploited. No product was formed by the replacement of the phenyl moiety of pyrazolidinone **20** with a pyridyl group. The current procedure showed high functional-group tolerance and great efficiency, providing a variety of corresponding compounds **22** in moderate to good yields under mild conditions [59].

### 2.2. Synthesis of Indazoles Using Ruthenium Salt in the Presence of Sodium Acetate

**Scheme 8:**
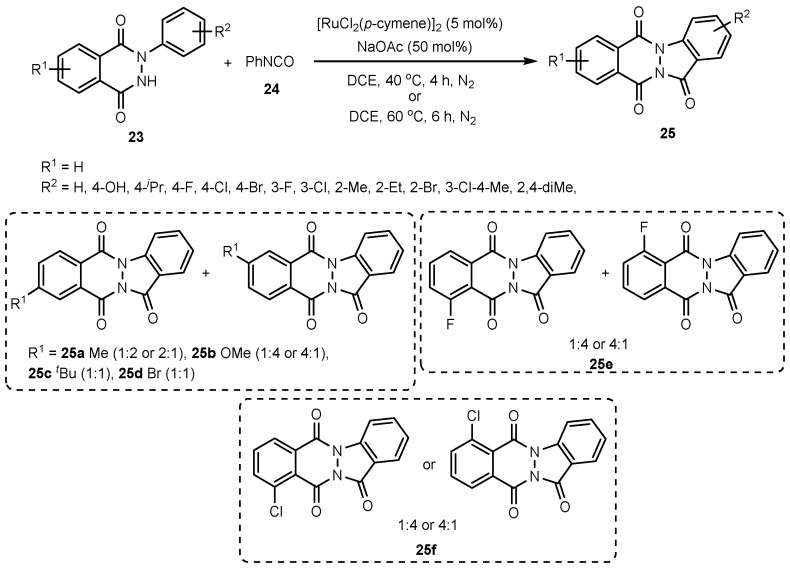
A synthetic route for the preparation of indazolo[1,2-*b*]phthalazine-trione derivatives **25** from the reaction between 2-aryl-2,3-dihydrophthalazine-1,4-diones **23** and isocyanates **24** under ruthenium catalysis.

Ru(II)-catalyzed tandem ortho-carbonylation/amidation/cyclization of 2-aryl-2,3-dihydrophthalazine-1,4-diones **23** has been developed towards the direct assembly of substituted indazolo[1,2-*b*]phthalazine-triones **25**. The reaction conditions involve [RuCl_2_(*p*-cymene)]_2_ (5 mol%) and NaOAc (50 mol%) in DCE as a solvent at 40 °C within 4 h under N_2_ atmosphere (Scheme 8). The scope and limitations of this tandem ortho-carbonylation/amidation/cyclization strategy with respect to 2-aryl-2,3-dihydrophthalazine-1,4-diones **23** have been explored. In general, good to high yields of the corresponding products were achieved by using meta- and para-substituted substrates **23** with both electron-rich, electron-poor, and halogen functional groups. However, the substrates **23** substituted at the ortho site underwent this tandem reaction only under harsh reaction conditions (DCE at 120 °C for 6 h) to afford the products in moderate to high yields. Plus, disubstituted substrates **23** with substituents located at the meta- and para-sites were amenable to give the desired products in DCE at 40 °C within 4 h, although the substrate with substituents located at the ortho- and para-sites gave the targeted product in DCE only after heating at 120 °C for 6 h. In the case of 6/7-substituted phthalazine derivatives (phthalazine scaffold with 6/7-Me, 6/7-MeO, 6/7-*t*-butyl, 6/7-Br, 6/7-F, and 6/7-Cl), mostly inseparable mixtures of the corresponding products **25a–f** were obtained. Indeed, a mixture of products was obtained, as a mixture of the starting materials was applied in the reaction.

Phenylhydrazines containing 5-methyl, 5-methoxy, 5-*t*-butyl, 5-bromo, and 4-fluorophthalic anhydrides provided inseparable mixtures of 6/7-methyl, 6/7-methoxy, 6/7-*t*-butyl, 6/7-bromo, and 5/8-fluoro-2-phenyl-2,3-dihydrophthalazine-1,4-diones **25a–e**, while phenylhydrazine condensation products with 4-chlorophthalic anhydrides provided 5/8-chloro-2-phenyl-2,3-dihydrophthalazine-1,4-dione **25f** as a single regioisomer. The amino, nitro, trifluoromethyl, or cyano functional groups on aryl/phthalazinone moieties did not work under the optimized reaction conditions [21].

**Scheme 9:**
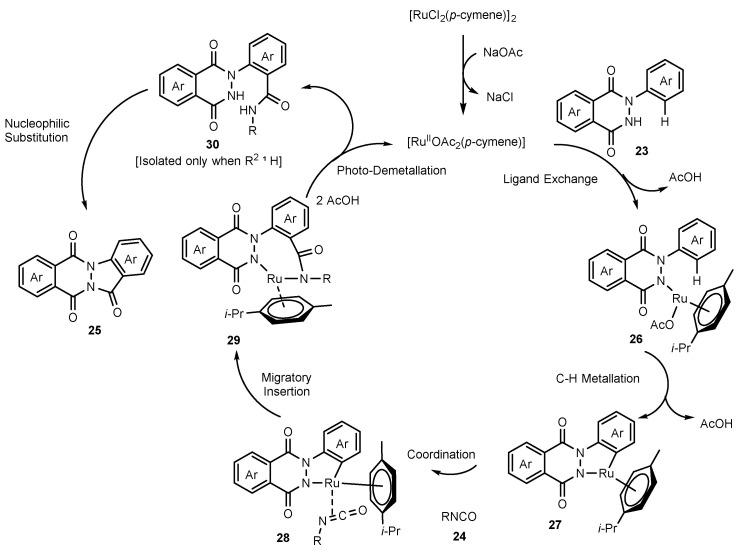
A rational mechanism for the synthesis of indazolo[1,2-*b*]phthalazine-trione derivatives **25** from the reaction between 2-aryl-2,3-dihydrophthalazine-1,4-diones **23** and isocyanates **24** under ruthenium catalysis.

A plausible mechanism for this reaction is illustrated in Scheme 9. Initially, an active catalyst was formed in situ from [RuCl_2_(*p*-cymene)]_2_ using NaOAc. The monomeric [Ru^II^OAc_2_(*p*-cymene)] species transferred to **26** via a ligand exchange with N-H of **23**, followed by C−H metalation to deliver a five-membered ruthenacyclic complex **27**. The catalytic center was then coordinated by the C=N double bond of aryl isocyanate **24**. Next, the migratory insertion of –C=N into a Ru-Ar bond gives a seven-membered ruthenacyclic complex **29**. The protonolysis of the two nitrogens in intermediate **29,** followed by an intramolecular nucleophilic substitution, provided a tetracyclic carbocyclized product **25** [21].

### 2.3. Synthesis of Indazoles Using Rhodium Complexes and Silver Salts

**Scheme 10:**
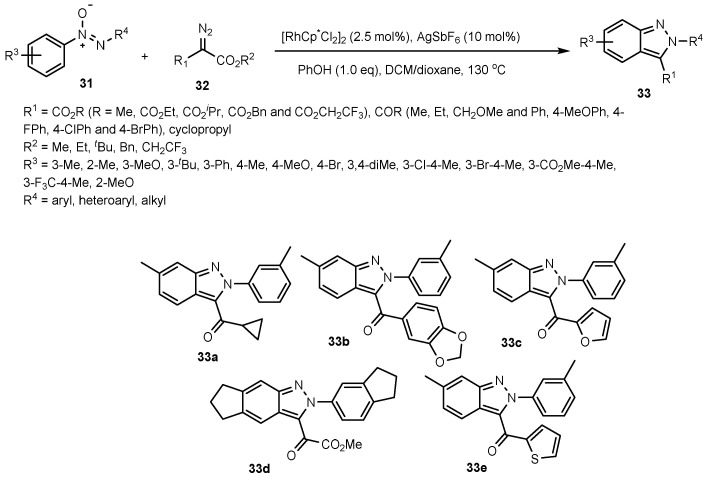
A synthetic route for the preparation of 2*H*-indazoles derivatives **33** via the regio- and chemoselective [4 + 1] annulation of azoxy compounds **31** with diazoesters **32** under rhodium catalysis.

An interesting methodology for the synthesis of 3-acyl-2*H*-indazoles **33** was performed using a Rh(III)-catalyzed tandem C–H alkylation/intramolecular decarboxylative cyclization of azoxy compounds **31** with diazoesters **32**. The combination of catalytic amounts of [Cp*RhCl_2_]_2_/AgSbF_6_ in the presence of PivOH in DCE/dioxane plays a crucial role to afford the corresponding products at 130 °C within 24 h. In this research, azoxy scaffold 31 was introduced as an incorporated directing group for regio- and chemoselective [4 + 1] cyclization, which could be promoted by diverse symmetrical and unsymmetrical di- and mono-aryldiazene oxides. The feasibility of the coupling of symmetrical and unsymmetrical di- and mono-aryldiazene oxides with diazoesters **32** was explored under standard conditions. First, several diazoesters **32** were investigated, affording *H*-indazoles. While the diisopropyl 2-diazomalonate led to a high yield, diazoester containing *t*-Bu led to a moderate yield, assumably owing to the easy hydrolysis of this functional group under the optimized reaction conditions. Moreover, *α*-diazo-*β*-keto esters participated in this annulation to the corresponding products in good to high yields. Generally, a wide range of functional-group tolerance for both *α*-diazo-*β*-alkyl keto esters and *α*-diazo-*β*-(hetero)aryl keto esters highlights this strategy for the regio- and chemoselective synthesis of 2*H*-indazoles. However, when diazoesters with *N*-heterocycles, including methyl 2-diazo-3-oxo-3-(pyridin-2-yl)propanoate, methyl 2-diazo-2-(pyridin-2-yl)acetate, and methyl 2-(benzo[d]thiazol-2-yl)-2-diazoacetate, were used under optimized reaction conditions, no product was formed. The diazo reagents including EtO_2_C-CHN_2_, EtO_2_C-C(CH_3_)N_2_, EtO_2_C-CPhN_2_, and EtO_2_C-C(CF_3_)N_2_ also did not work with this catalytic system. While symmetrical azoxybenzenes substituted at the ortho, meta, and para-positions of aryl rings could be applicable in this reaction, azoxybenzenes substituted at the meta-position of aryl rings delivered a fairly low yield, conceivably because of steric congestion. The azoxybenzenes substituted at the ortho-position could be exploited as coupling partners, with coupling occurring exclusively in the sterically less hindered site. Some more complex molecules **33a–e** are shown in Scheme 10. The advantages and benefits of this transformation are regioselectivity for unsymmetrical azoxybenzenes and the compatibility of monoaryldiazene oxides [60].

**Scheme 11:**
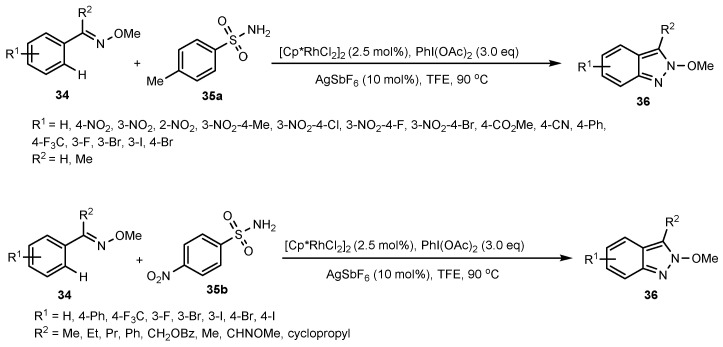
A synthetic route for the preparation of indazoles derivatives **36** via the oxidative annulation of ketoxime methyl ethers **34** with sulfonamide **35a–b** under rhodium catalysis.

The rhodium(III)-catalyzed synthesis of indazole derivatives **36** has been realized through an intermolecular C–H amination and N–N bond formation sequence starting from readily available ketoxime ethers **34** and 4-toluenesulfonamide **35a** (Scheme 11). All reactions were carried out using 2.5 mol% of [Cp*RhCl_2_]_2_ as a catalyst, 0.6 mmol PhI(OAc)_2_, and 10 mol% of AgSbF_6_ in trifluoroethanol (TFE) at 90 °C for 24 h. First, the functional-group tolerance of the ketoxime derivatives **34** was explored under optimized reaction conditions. Although the reaction of meta- or para-nitro-substituted acetophenone oxime derivatives was successfully carried out to afford moderate to good yields of desired products, the 2-nitrobenzaldehyde oxime methyl ether only gave the corresponding indazole **36** in a moderate yield due to steric effects on the aryl ring. The unsubstituted acetophenone oxime derivatives were all suitable for this system, leading to desired indazoles in satisfactory yields. In the next step, both *p*-ester- and cyano-substituted acetophenone oxime ethers were proven to be appropriate substrates for this transformation as well. The acetophenone oxime derivative exhibited less efficacity in this oxidative annulation, affording the expected indazole in a 15% yield. Afterward, the authors investigated more amides to address the low reactivity of 4-toluenesulfonamide **35a** in this reaction. The results showed that phenylsulfonamides **35b** containing electron-deficient substituents produced better yields of the target indazoles **36**. Several substituted acetophenone oxime ethers **34** derived from propiophenone, *n*-butyrophenone, cyclopropyl phenyl ketone, and diphenylketone exhibited good compatibility for the reaction with 4-nitrobenzenesulfonamide **35b**. Notably, substituents such as F, Br, I, and CF_3_ on the aromatic ring of the acetophenone oxime ethers all survived the reaction conditions, affording the desired products **36** in good yields. To sum up, a broad range of substituents were possible with this catalytic system, producing different functionalized indazoles with acceptable yields [61].

**Scheme 12:**
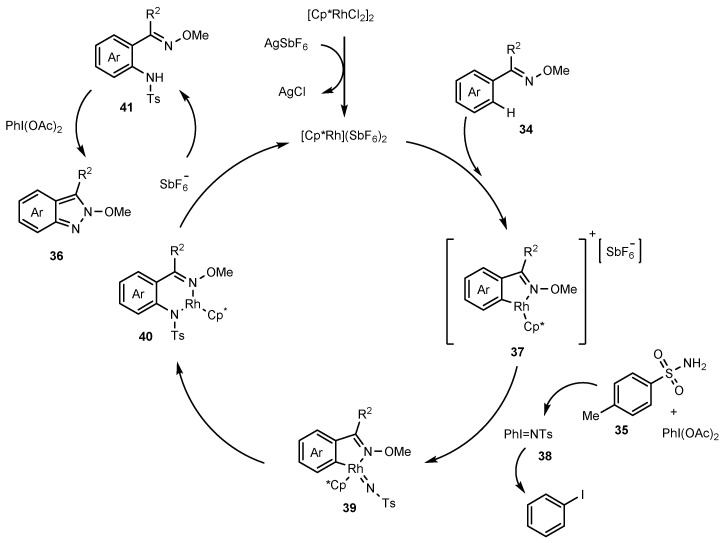
A possible mechanism for the synthesis of indazole derivatives **36** via the oxidative annulation of ketoxime ethers **34** with sulfonamide **35** under rhodium catalysis.

The mechanism involves (Scheme 12) the electrophilic activation of Rh by chloride removal with the silver salt, the coordination of ketoxime ether **34** with an active catalyst, and C−H activation, respectively, to form the complex **37**. This complex is oxidized in the presence of in situ-generated iodonium **38** to give the complex **39**. In the next step, migratory insertion and releasing [Cp*Rh](SbF_6_)_2_ as the active complex occurs to form **41**. Finally, target product **36** is obtained via the reaction with iodobenzene diacetate [61].

### 2.4. Synthesis of Indazoles Using Rhodium and Silver Salts in the Presence of Lithium Acetate

**Scheme 13:**
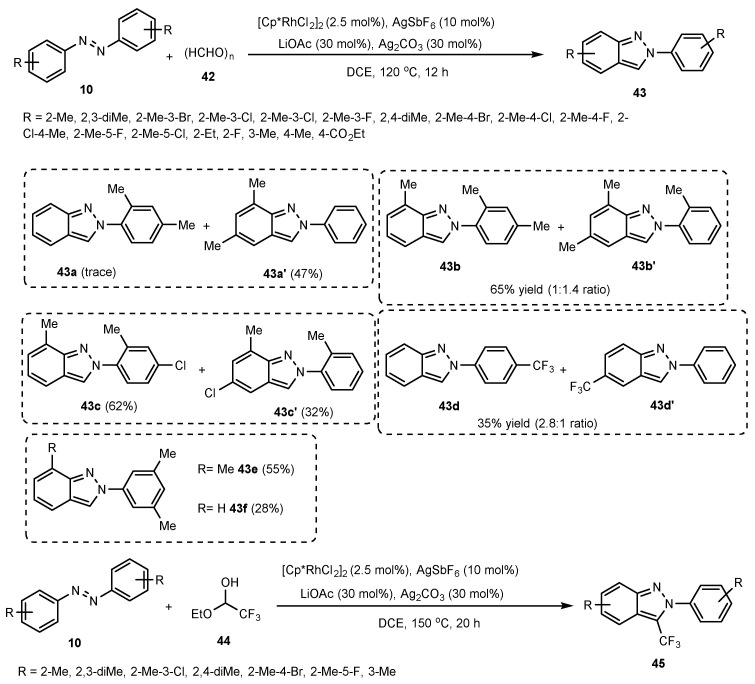
A synthetic route for the preparation of 1*H*-indazole derivatives **43** and **45** from azobenzenes **10** using paraformaldehyde **42** as a one-carbon synthon or trifluoroacetaldehyde ethyl hemiacetal **44**.

The synthesis of C3-unsubstituted and C3-trifluoromethylated (2*H*)-indazoles starting from azobenzenes **10** and paraformaldehyde **42** as a useful C1-feedstock under rhodium catalysis was reported by Kim et al. in 2019. The strategy was also successfully expanded to C−H activation/annulation reactions of azobenzenes **10** with trifluoroacetaldehyde hemiacetal **44**. The optimized conditions for the synthesis of (2*H*)-indazoles **43** and **45** from azobenzenes **10** included 2.5 mol% of [RhCp*Cl_2_]_2_, 10 mol% of AgSbF_6_, 30 mol% of LiOAc, and 30 mol% of Ag_2_CO_3_ in DCE as a solvent at 120 °C for 12 h (or 150 °C for 20 h) under air atmosphere in sealed reaction tubes. LiOAc and AgSbF_6_ were used to activate the Rh catalyst by forming [Cp*Rh(III)(OAc)]SbF_6_. Various symmetrical ortho-, meta-, and para-disubstituted azobenzenes underwent coupling, with paraformaldehyde **42** showing good functional-group tolerance. While symmetrical 2,5-disubstituted azobenzenes (5-fluoro-2-methyl azobenzene and 5-chloro-2-methyl azobenzene) were well-tolerated and the desired products were obtained in 58% and 12% yields, respectively, sterically congested 2,5-dimethyl azobenzene failed to afford the corresponding product. In the next step, several unsymmetrical azobenzenes were screened as substrates, and the products **43a–d** and **43a’–43d’** were obtained (Scheme 13). Notably, the steric environment of the azobenzene orients the formation of desired products **43e–f**. The substrate scope of this reaction was further expanded to trifluoroacetaldehyde ethyl hemiacetal **44** to produce a range of C3-CF_3_-substituted (2*H*)-indazoles **45** in moderate to high yields. This conversion efficiently afforded several C3-unsubstituted and C3-trifluoromethylated (2*H*)-indazoles **43** and **45**, which are important molecules in organic chemistry and pharmaceutical sciences. The practicability of this approach is highlighted by its chemoselectivity, functional-group tolerance, and wide substrate scope [62].

### 2.5. Synthesis of Indazoles Using Rhodium and Silver Salts in the Presence of Sodium Acetate

**Scheme 14:**
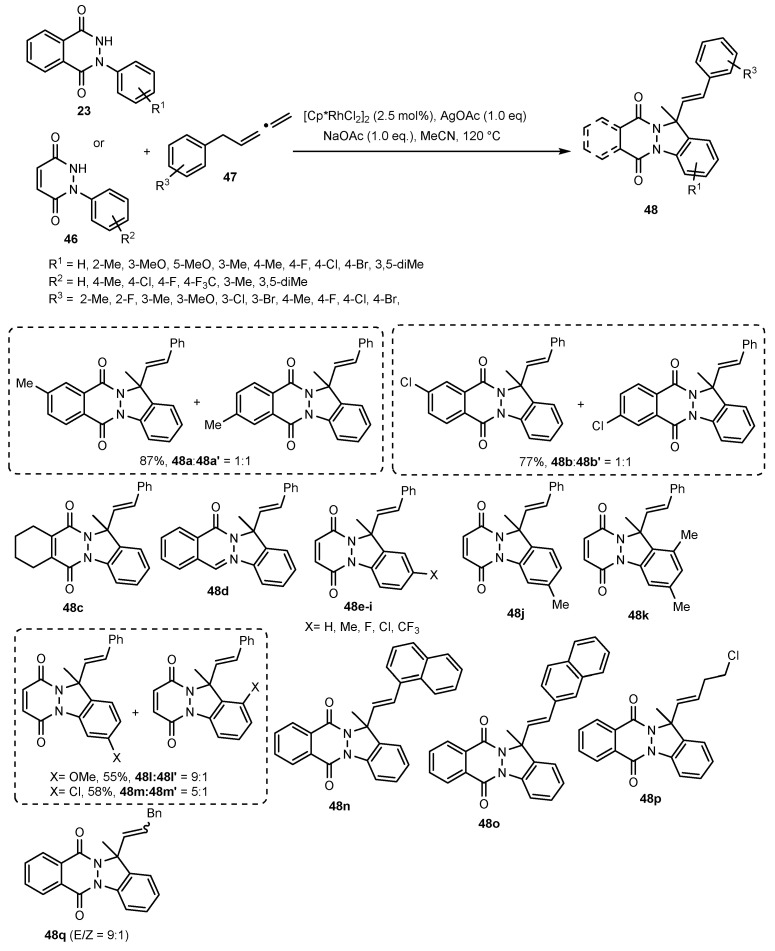
A synthetic route for the preparation of indazole derivatives **48** through the C–H activation of phthalazinones **23** or pyridazinones **46** and allenes **47** under Rh(III) catalysis.

Yu’s group developed an efficient and novel Rh(III)-catalyzed annulation transformation between phthalazinones/pyridazinones **23** and **46** and different allenes **47** into indazole derivatives **48** containing a quaternary carbon (Scheme 14). A C–H activation and olefin insertion sequence, followed by *β*-hydride elimination and intramolecular annulation occurs to produce indazole derivatives **48** using a catalytic amount of [Cp*RhCl_2_]_2_ in the presence of AgOAc in MeCN at 100 °C within 12 h under air atmosphere. The active Rh catalyst is formed in situ by the ion exchange of chloride to acetate involving [Cp*RhCl_2_]_2_ and AgOAc/NaOAc. In other words, acetate ions replace chloride ligands to give [Cp*Rh(OAc)_2_]_2_. *N*-Aryl phthalazinone and pyridazinone substrates bearing a range of electron-rich electron-poor substituents at different positions could deliver the corresponding products in satisfactory yields. The [4 + 1] cyclization of the substrate substituted by methyl at the ortho-position of the *N*-aryl affords the desired product in only a 26% yield because of steric hindrance. On the other hand, the reaction of the *N*-aryl substrate with methyl at the meta-position demonstrated remarkable reactivity and excellent chemoselectivity, while the corresponding meta-methoxy analogs gave an isomeric mixture in a 9:1 ratio. Para-substituted *N*-aryl phthalazinone substrates **23** with a variety of electron-rich and electron-poor functional groups can be easily transformed into the desired indazoles **48** in good to high yields under standard reaction conditions. It is worth noting that the substrate substituted by two methyl groups at the 3 and 5 positions of the *N*-aryl segment worked well to give the corresponding product in a good yield. The products **48a** and **48b** could be formed with moderate selectivity (1:1). The products **48c** and **48d** were also obtained under standard conditions. The target compounds **48e–m** were also achieved via this [4 + 1] cyclization in good yields. The substrate scope of a variety of allenes **47** substituted by several electron-donating or electron-deficient groups at different positions gave the corresponding products in acceptable to high yields. 1-Naphthyl- or 2-naphthyl-group-substituted allenes and aliphatic allene were also well-tolerated under these reaction conditions, thus producing the corresponding indazoles **48n–q** in good to high yields. Notably, a long-chain aryl-substituted substrate yielded the desired product **48q** with good selectivity (*E/Z* = 9:1) in a high yield. Finally, 1,1,4-trisubstituted and simple aliphatic allenes containing an ester group did not work under optimized reaction conditions. The approach successfully tolerates different substituents on the starting phthalazinones/pyridazinones and allenes and features practicability, high atom efficiency, and high Z-selectivity [63].

**Scheme 15:**
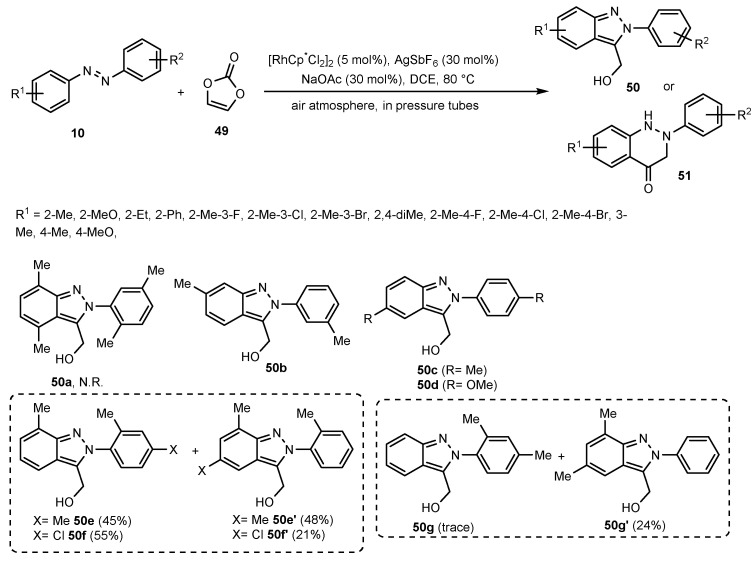
A synthetic route for the preparation of 2*H*-indazole derivatives **50** via the annulation of azobenzenes **10** with vinylene carbonate **49** under Rh(III) catalysis.

The synthesis of C3-hydroxymethylated (2*H*)-indazoles **50** can be achieved through a subsequential Rh(III)-catalyzed C–H functionalization/intramolecular cyclization between azobenzenes **10** and vinylene carbonate **49** using catalytic amounts of [RhCp*Cl_2_]_2_, AgSbF_6_, and NaOAc in DCE at 80 °C within 14 h under air atmosphere in pressure tubes (Scheme 15). The electronic properties of the azobenzene rings can orient towards the construction of (2*H*)-indazoles **50** or the isomeric dihydrocinnolin-4-ones **51**. It is worth noting that the vinylene carbonate substrate plays a significant role in the promotion of the [4 + 1] or [4 + 2] cyclization. The substrate scope and limitations of the azobenzenes **10** were tested under optimized reaction conditions. The coupling transformations were successful for ortho-substituted azobenzenes containing electron-donating substituents, obtaining moderate to high yields of C3-hydroxymethylated (2*H*)-indazoles. This catalytic system allows moderate functional-group tolerance, particularly for –F, –Cl, and –Br substituents. No corresponding product **50a** was formed by the utilization of sterically congested 2,5-dimethyl-substituted azobenzene. Meta- and para-methyl substituted azobenzenes were also applicable using this catalytic system, although the desired products **50b** and **50c** were obtained with relatively low yields. Moreover, para-OMe azobenzene was proven to be an efficient substrate, producing **50d** in good yield. This exploration was further extended to the coupling of unsymmetrical azobenzenes with vinylene carbonate **49**. For example, azobenzenes containing electron-donating substituents on both aryl rings afforded almost equimolar ratios of **50e** and **50e’** under standard conditions. In the case of other unsymmetrical azobenzenes, the transformation mainly occurred on the more electron-donating aromatic ring, as for **50f** (**50f’**) and **50g** (**50g’**). Furthermore, it was shown that no C3-hydroxymethylated (2*H*)-indazole was obtained using azobenzenes containing electron-poor substituents, and only 2,3-dihydrocinnolin-4-ones **51** were formed via [4 + 2] annulation transformation. When unsubstituted azobenzene was used as a substrate in the reaction, a mixture of both (2*H*)-indazole and 2,3-dihydrocinnolin-4-one was produced. The notable benefits of this methodology are mild reaction conditions and excellent functional group compatibility, thus furnishing easy access to a series of indazoles of interest in organic chemistry and pharmacy [64].

**Scheme 16:**
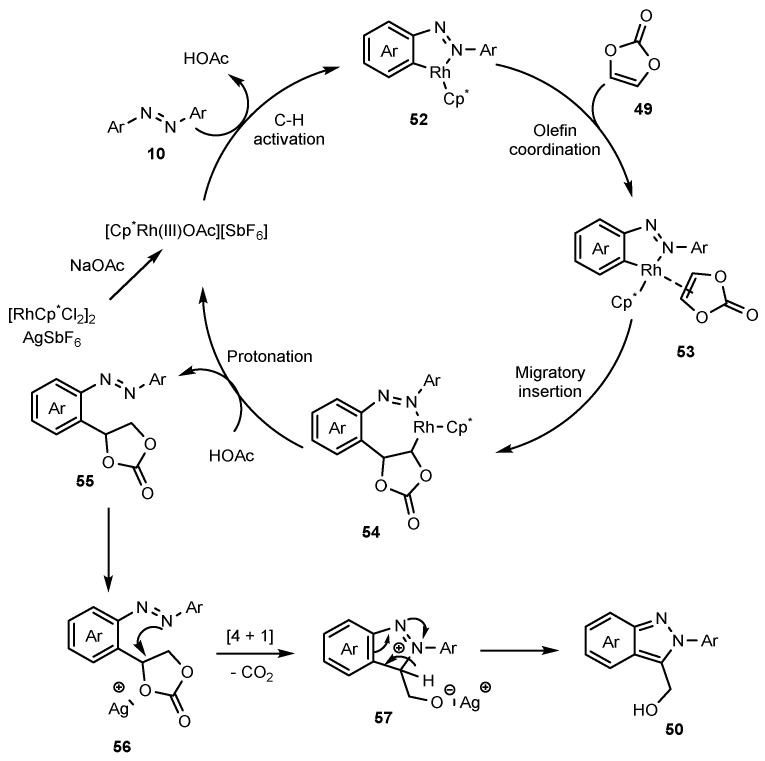
A possible mechanism for the synthesis of 2*H*-indazole derivatives **50** via the annulation of azobenzenes **10** with vinylene carbonate **49** under Rh(III) catalysis.

The mechanism for the synthesis of (2*H*)-indazoles **50** via the annulation of azobenzenes **10** with vinylene carbonate **49** under Rh(III) catalysis is illustrated in Scheme 16. First, the active catalyst is formed from [RhCp*Cl_2_]_2_ and AgSbF_6_ in the presence of NaOAc. The C−H bond of azobenzene **10** is activated to deliver a five-membered rhodacycle intermediate **52**. Subsequently, complex **52** undergoes olefin coordination, migratory insertion, and protonation to afford the ortho-alkylated compound **55**. In the next step, Ag^+^ acts as a Lewis acid to activate the nucleophilic substitution of an azo group at the *α*-position on the dioxolan-2-one scaffold, followed by an aromatization transformation to produce (2*H*)-indazole **50** [64].

### 2.6. Synthesis of Indazoles Using Rhodium and Silver Salts in the Presence of Zinc Acetate

**Scheme 17:**
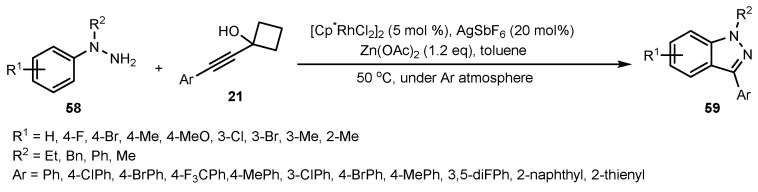
A synthetic route for the preparation of 1*H*-indazoles **59** via hydrazine-directed C−H functionalization with 1-alkynylcyclobutanols **21**.

The commercially available [Cp*RhCl_2_]_2_ has been exploited as a catalyst for the reaction of phenylhydrazines **58** with 1-alkynylcyclobutanols **21** in the presence of SbF_6_ and Zn(OAc)_2_ in toluene at 50 °C for 24 h under Ar atmosphere (Scheme 17). Apparently, the reaction proceeds via a hydrazine-directed C–H functionalization process. This catalytic system provided an efficient protocol to produce 1*H*-indazole derivatives **59** via [4 + 1] annulation based on the cleavage of an alkyne bond with good functional group compatibility and moderate to good yields. First, the substrate scope of 1-arylethynyl cyclobutanols **21** was investigated, and all the results showed that 1-arylethynyl cyclobutanol derivatives containing both electron-deficient and electron-rich substituents on the para- or meta-sites of the benzene ring could afford the corresponding indazoles **59** in acceptable yields. It was found that di-fluoro or 2-thienyl substituted substrates could deliver the corresponding indazole in satisfactory yields using AgNO_3_ instead of AgSbF_6_. In the next step, several arylhydrazines substituted at the position of the aryl-linked nitrogen were screened. While 1-ethyl and 1-benzyl phenylhydrazines delivered the corresponding indazoles **59** in good yields, the *N*-phenyl substituted substrate did not work in this reaction, presumably because of its higher steric hindrance. Arylhydrazines containing halides such as F, Cl, and Br as well as electron-rich substituents such as Me and OMe provided the desired products in satisfactory yields. Notably, 1-alkyl-1-phenylhydrazines substituted at the ortho-position were compatible under optimized reaction conditions [65].

**Scheme 18:**
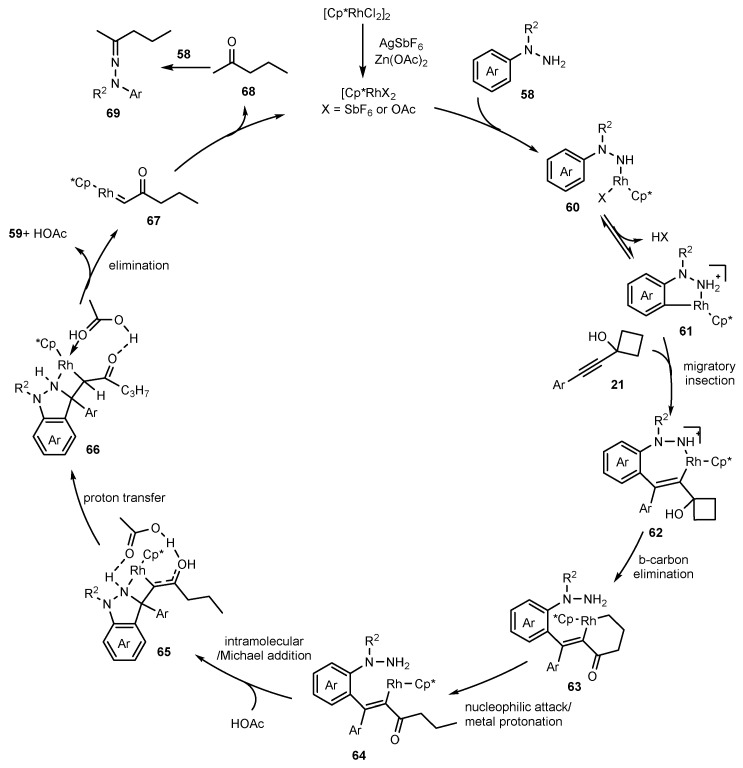
A plausible mechanism for the synthesis of 1*H*-indazoles **59** via hydrazine-directed C−H functionalization with 1-alkynylcyclobutanols **58**.

The first step of the mechanism comprises the activation of a rhodium catalyst using AgSbF_6_/Zn(OAc)_2_. In the case of Zn(OAc)_2_, apparently the zinc ion itself plays no role, and only the acetate ion activates the Rh catalyst (Scheme 18). Then, the hydrazine group of **58** coordinates to the metal center of this active complex. Next, C(aryl)−H activation, regioselective migratory insertion, and *β*-carbon elimination happen to form a six-membered rhodacycle intermediate **63**. In the next step, an intramolecular nucleophilic attack of phenyl hydrazine nitrogen on rhodium(III) ion affords intermediate **64**. Finally, the target molecule **59** is formed via an intramolecular Michael addition, a proton transfer, and releasing a rhodium-carbene **67** [65].

### 2.7. Synthesis of Indazoles Using Rhodium and Silver Salts in the Presence of Magnesium Sulfate

**Scheme 19:**
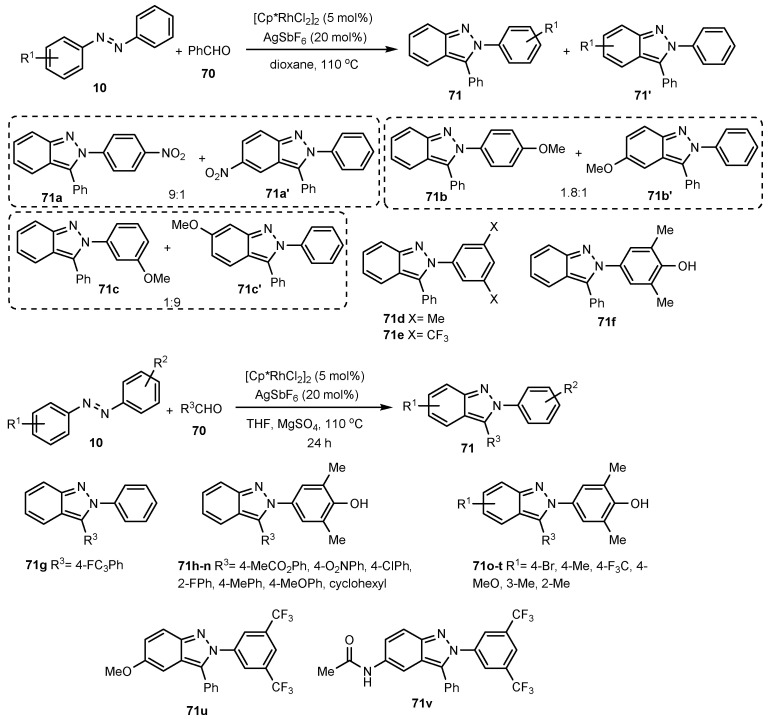
A synthetic route for the preparation of indazole derivatives **71** via C−H bond functionalization and cyclative capture.

A convenient transformation for the construction of substituted *N*-aryl-2*H*-indazole derivatives **71** through the reaction of azobenzenes **10** with aldehydes **70** in a simple, efficient, and one-step process was reported by Ellman et al. in 2013 (Scheme 19). The reactions proceeded well to give the corresponding indazoles **71** by Rh(III)-catalyzed C–H bond functionalization and cyclative capture. The reaction of azobenzene with benzaldehyde was promoted by 5 mol% (Cp*RhCl_2_)_2_ and 20 mol% AgSbF_6_ in dioxane at 80 °C for 24 h. Whereas the unsymmetrical para-nitro azobenzene provided major product **71a** by annulation on the more electron-rich phenyl ring of the azobenzene, 4-methoxy-substituted azobenzene produced a mixture of products **71b** and **71b′** in low selectivity in which a C−H activation/annulation sequence occurred on the less electron-donating phenyl ring of the azobenzene. In contrast to para-nitro substituted azobenzene, the 3-methoxy substituted substrate afforded a mixture of products with 9:1 regioselectivity, favoring **71c′** with C−H activation on the more electron-donating phenyl ring. This fact displayed the capacity of meta-MeO for supplying resonance stabilization. The high regioselectivity observed for the synthesis of products **71d–f** indicated that steric effects are important. In the case of hydroxy-substituted azobenzene, the yield of product **71f** could be improved by the utilization of MgSO_4_ as a drying agent and THF as a solvent. Next, aromatic aldehydes bearing both electron-deficient and electron-donating substituents proved to be suitable in the reaction with a 4-hydroxy-3,5-dimethylphenyl azobenzene substrate, leading to good to high yields of products **71h–v**. Notably, aliphatic aldehyde provided the desired indazole **71n** in a low yield. This reaction offers remarkable advantages, including the simplicity of the reaction, excellent regioselectivity, good functional-group tolerance, and the exploitation of commercially available materials [66].

### 2.8. Synthesis of Indazoles Using Ruthenium and Copper Salts in the Presence of Potassium Hexafluorophosphate

**Scheme 20:**
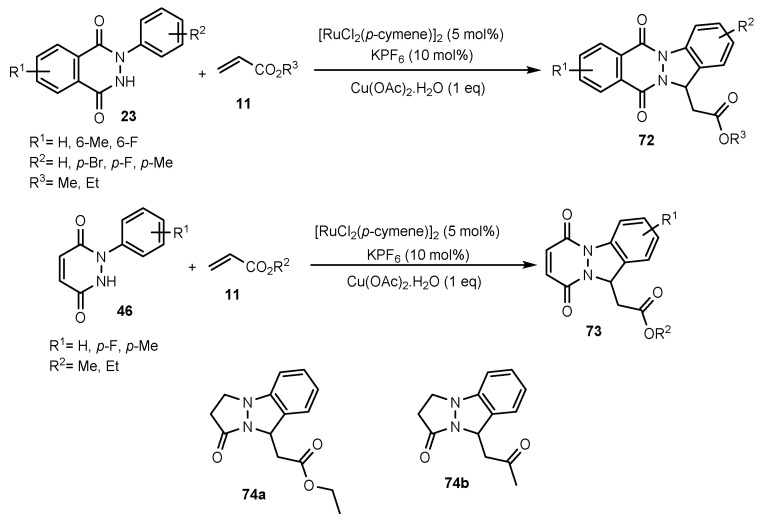
A synthetic route for the preparation of indazole derivatives **72**, **73,** and **74** via an alkenylation–annulation approach.

A Ru-catalyzed cascade reaction between *N*-aryl phthalazinediones **23** or *N*-aryl pyridazinediones **46** with acrylates proceeded through a subsequential oxidative alkenylation/intramolecular cyclization in aqueous media as a green solvent (Scheme 20). All reactions proceeded successfully in the presence of [RuCl_2_(p-cymene)]_2_ (5 mol%) as a catalyst, KPF_6_ (10 mol%) as an additive, and Cu(OAc)_2_·H_2_O (1 eq) as an oxidant to provide moderate to high yields of products. The limitations and diversity of this alkenylation–annulation via C–H bond activation were explored with respect to *N*-aryl phthalazinedione **23** and *N*-aryl pyridazinediones **46** derivatives. *N*-Aryl phthalazinediones **23** and *N*-aryl pyridazinediones **46** containing electron-deficient or electron-rich substitutions on the aromatic rings afforded the desired indazole derivatives **72** with moderate to excellent yields. The reaction conditions did not work for methyl methacrylate. Moderate yields of desired products **74a** and **74b** were achieved by using substrates bearing a pyrazolidinone scaffold [67].

**Scheme 21:**
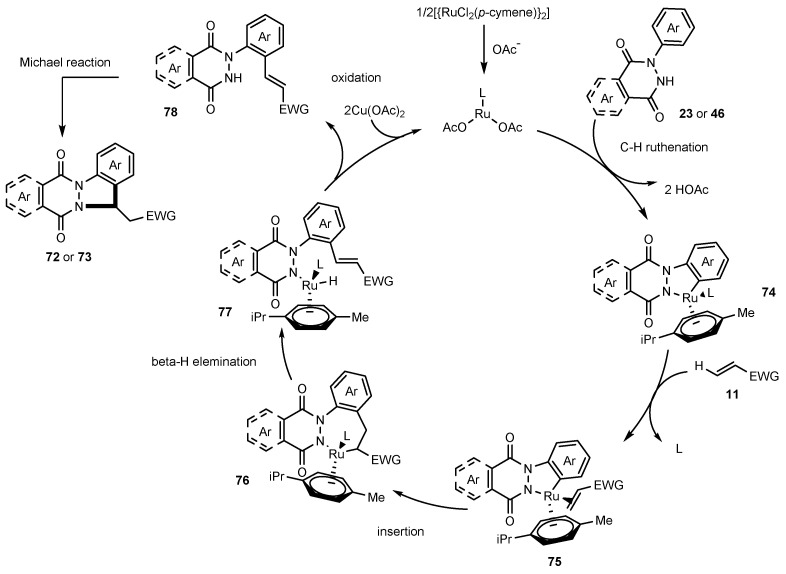
A plausible mechanism for the synthesis of indazole derivatives **72** or **73** via an alkenylation–annulation approach.

Subsequent oxidative vinylation and annulation are the key steps for this transformation. A possible mechanism for the reaction is shown in Scheme 21. The transformation is initiated by N–H assisted C–H bond ruthenation of compound **23** or **46** to construct a ruthenacycle complex **74**. Next, migratory insertion, *β*-hydride-elimination, and reductive elimination occurred to form alkenylated product **78**. Eventually, an intramolecular aza-Michael addition reaction took place to produce the target indazoles **72** or **73** [67].

### 2.9. Synthesis of Indazoles Using Rhodium, Copper, and Silver Salts

**Scheme 22:**
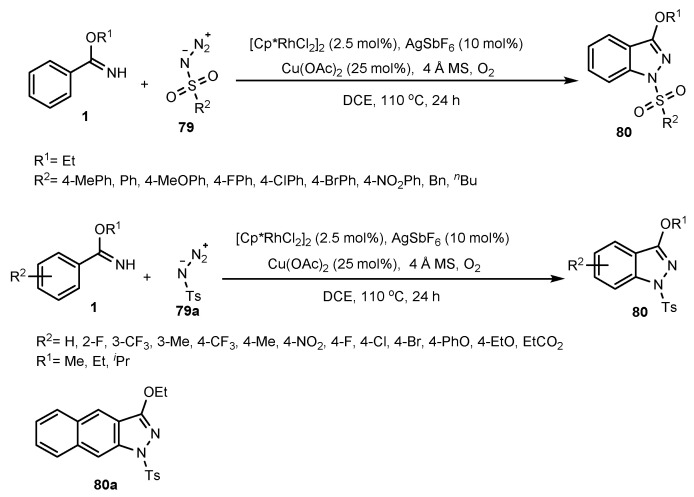
A synthetic route for the preparation of 1*H*-indazole derivatives **80** via C−H amidation and N−N bond formation.

In 2013, Glorius and co-workers developed a process involving Rh(III)-catalyzed C–H activation/C–N bond formation and Cu-catalyzed N–N bond formation under mild reaction conditions for the synthesis of substituted 1*H*-indazoles **80** using [Cp*RhCl_2_]_2_, Cu(OAc)_2_, and AgSbF_6_ catalysts and molecular oxygen as the oxidant (Scheme 22). For the first time, in this study, it was possible to perform the transformation of N-H-imidates **1** to corresponding compounds **80**. Diverse arylsulfonyl azide derivatives **79** with 4-Me, 4-OMe, 4-NO_2_, 4-F, 4-Cl, and 4-Br as substituents as well as alkylsulfonyl azides **79** (R_2_ = Bn, Bu) demonstrated moderate to high reactivity in this conversion. Several arylimidates were exploited to explore the reactivity of different substituents in the alkoxy and arene parts. The ethylimidate **1** (R_4_ = OEt) revealed a more satisfactory yield than methyl **1** (R_4_ = OMe) and isopropyl **1** (R_4_ = O-*i*-Pr) derivatives. Contrary to the electron-donating arylimidates, the electron-withdrawing imidates afforded better yields for this transformation. This cascade reaction is practical, scalable, and green, using O_2_ as the stoichiometric oxidant. In addition, only N_2_ and H_2_O are the byproducts of this reaction. It is worth noting that indazole was formed with a <5% yield when omitting either of the Rh or Cu catalysts for the model reaction, denoting the important role of these two catalysts in the mechanism cycle [68].

**Scheme 23:**
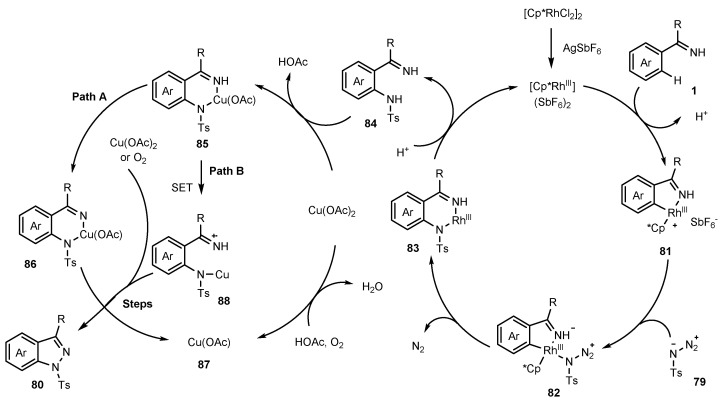
A rational mechanism for the synthesis of 1*H*-indazole derivatives **80** via C−H amidation and N−N bond formation.

Mechanistically, the construction of indazoles **80** comprises the steps shown below in Scheme 23. First, the active catalyst (cationic [Cp*Rh^III^]) is obtained in situ using AgSbF_6_ to remove the chloride. Subsequently, coordination to imidate **1** and C−H activation are performed by the active catalyst to give rhodacyclic complex **81**. The coordination of azide substrate **79** to the catalyst followed by nitrogen loss and migratory insertion yields Rh^III^ amido species **83**. The resulting intermediate **83** is protonated to regenerate the active catalyst and provide the amidated compound **84**. In the next step, the resulting compound may coordinate to the copper source to form intermediate **85**. The authors considered two paths for the continuation of the mechanism. In route A, the Cu^II^ complex is transferred to a higher valent Cu^III^ complex in the presence of another molecule of Cu(OAc)_2_ or O_2_. Next, the construction of the N−N bond via reductive elimination formed the target compound **80**. In the final step, Cu^I^ complex **87** is transformed to Cu(OAc)_2_ in the presence of O_2_. As shown in Scheme 23, the alternative route **B** is able to produce a N−N bond through double single-electron transfer [68].

**Scheme 24:**
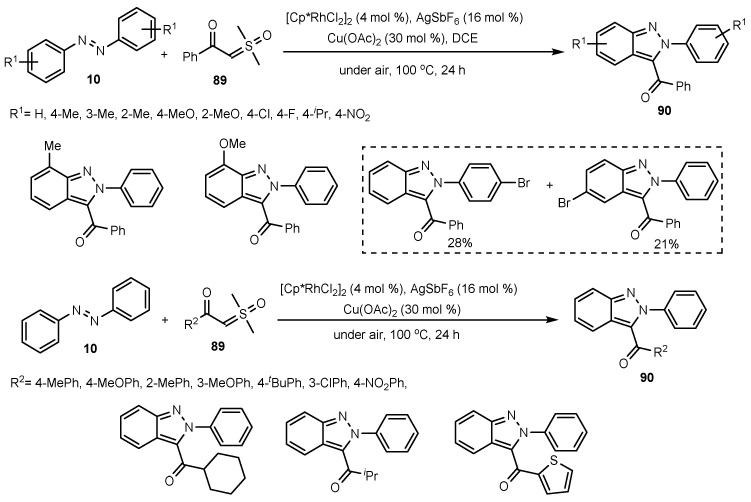
A synthetic route for the preparation of 3-Acyl-(2*H*)-indazoles **90** via the [4 + 1] annulation of azobenzenes **10** with *α*-carbonyl sulfoxonium ylides **89**.

An efficient strategy for the synthesis of 3-acyl-(2*H*)-indazoles **90** via an Rh(III)/Cu(II)-catalyzed [4 + 1] annulation of azobenzenes **10** with *α*-carbonyl sulfoxonium ylides **89** has been developed by Cheng et al. (Scheme 24). The sequential catalytic aromatic C–H bond activation and cyclization steps in which sulfoxonium ylides **89** served as efficient and stable carbene precursors are highly important in the chemical synthesis of the corresponding indazoles **90**. The model reaction produced 3-acyl-(2*H*)-indazole in a 20% yield in the absence of Cu(OAc)_2_ in DCE under air at 100 °C within 24 h. The scope and limitation of azobenzene derivatives **10** and *α*-carbonyl sulfoxonium ylide derivatives **89** as starting materials were explored, as illustrated in Scheme 24. Some functional groups on azobenzene rings, such as methyl, methoxy, Cl, F, *i*-Pr, and Br, were very compatible under the optimized reaction conditions with moderate to high yields. The reaction progressed efficiently by using sterically hindering substituents on the azobenzene ring under standard reaction conditions. As expected, with *m*-substituted substrates, the corresponding indazole derivatives were produced at the less hindered position. Unfortunately, the 4-nitro analogue of azobenzene failed to work. Unsymmetrical azobenzenes with Me and MeO groups were also explored, indicating the orientation of electron-rich aryl rings for the C−H activation and cyclization transformations. Next, sulfoxonium ylides with both electron-donating and electron-withdrawing substitutions were investigated. Several substitutions such as Me, MeO, Cl, NO_2_, and *t*-Bu as well as alkyl- and heteroaryl-substituted analogues were found to be appropriate under standard reaction conditions. The reaction displayed a broad substrate scope, moderate to excellent yields, and tolerance to various substituents [69].

**Scheme 25:**
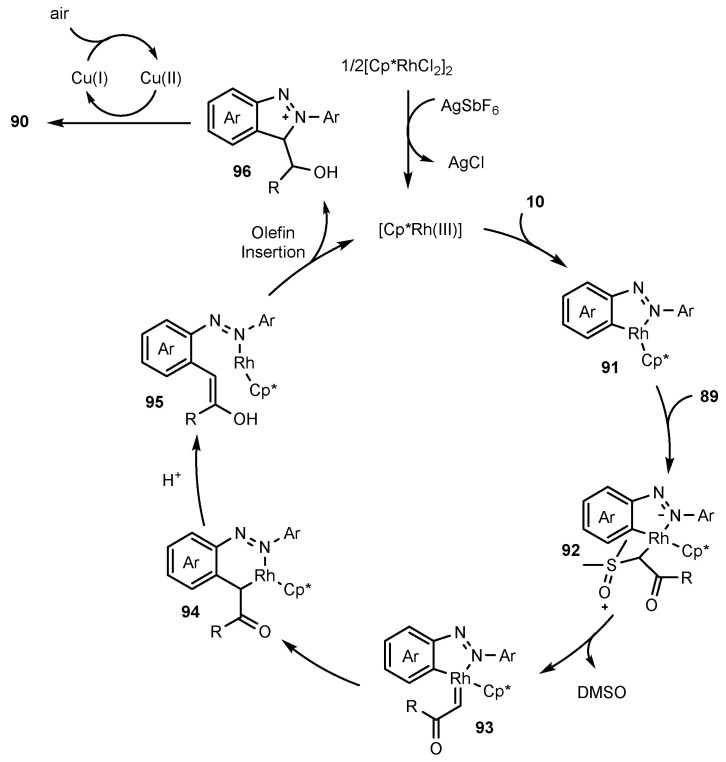
A plausible mechanism for the synthesis of 3-acyl-(2*H*)-indazoles **90** via the [4 + 1] annulation of azobenzenes **10** with *α*-carbonyl sulfoxonium ylides **89**.

Initially, the catalyst is activated in the presence of AgSbF_6_. Next, the Rh(III) catalyst activates the C−H bond of azobenzenes **10** to deliver five-membered cyclorhodium species **91**. Rh(III) intermediates **92** are generated by the insertion of sulfoxonium ylides **89** to the rhodium species. Six-membered rhodacycle species **94** are obtained via the *α*-elimination of DMSO followed by the migratory insertion of carbene complex **93**. The sequence of keto-enol tautomerization, the insertion of a C=C bond into the Rh-N bond, and the oxidation/aromatization in the presence of a copper catalyst produces **95**, **96,** and final products **90,** respectively (Scheme 25) [69].

**Scheme 26:**
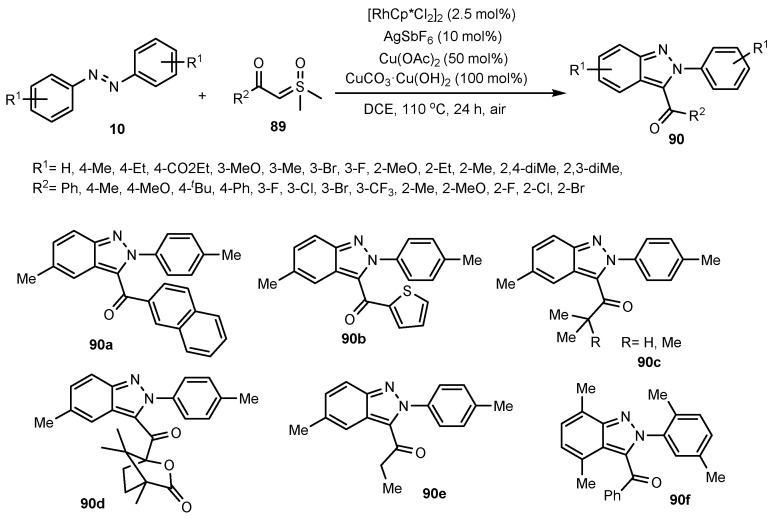
A synthetic route for the preparation of 2*H*-indazoles **90** through the annulation reaction of azobenzenes **10** with sulfoxonium ylides **89**.

The Rh(III)-catalyzed ortho-C−H bond activation of azobenzenes **10,** followed by the intramolecular annulation transformation of azobenzenes **10** between sulfoxonium ylides **89**, has been reported for the synthesis of 2*H*-indazole derivatives **90** in good to excellent yields with broad substrate scope, good selectivity, and good functional-group tolerance (Scheme 26). The combination of [RhCp*Cl_2_]_2_, AgSbF_6_, Cu(OAc)_2_, CuCO_3_·Cu(OH)_2_, and DCE at 110 °C for 24 h under air atmosphere in reaction tubes was found to be effective for this transformation. Next, the scope of these annulation reactions was examined, and the results are summarized in Scheme 26. The reactions using electron-rich para-substituted azobenzenes (such as Me, Et, and *t*-Bu) afforded the expected indazoles in excellent yields. Furthermore, the electron-rich meta- and ortho-substituted azobenzenes (such as OMe and Br at the meta-position or OMe, Me, and Et at the ortho-position) were explored, and a variety of indazoles were successfully produced in good to excellent yields. An electron-deficient azobenzene (CO_2_Et at the para-position) was also applicable to this reaction, which afforded the corresponding product in a moderate yield, while electron-deficient meta-substituted azobenzenes (Br and F in the meta-position) were proven to be relatively less efficient for this transformation. Although a sterically congested disubstituted azobenzene (two methyl groups at the 2- and 5-positions) did not give the desired product, by employing disubstituted azobenzenes (two methyl groups at the 2- and 4-positions or two methyl groups at the 2- and 3-positions) as substrates, the corresponding products were achieved in good yields. The steric congestion of product **90f** may be the reason of why 2,5-disubstituted azobenzene did not work in the reaction [70].

**Scheme 27:**
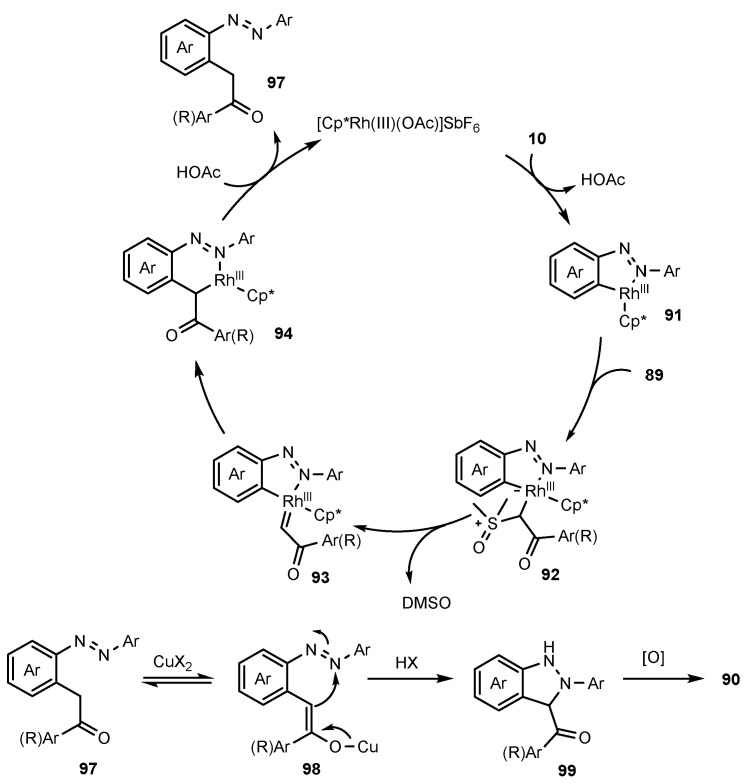
A possible mechanism for the synthesis of 2*H*-indazoles **90** through the annulation reaction of azobenzenes **10** with sulfoxonium ylides **89**.

A plausible reaction mechanism for the Rh(III)/Cu(II) catalyzed synthesis of indazoles **90** is shown in Scheme 27. The mechanism comprises the coordination of the azo group to the Rh(III) catalyst, C–H bond activation, the coordination of sulfoxonium ylides **89**, and the *α*-elimination of DMSO to give a reactive *α*-oxo Rh-carbene species **93**. In the next step, migratory insertion and, subsequently, protonation occur to provide **97**. Base-mediated intramolecular annulation and oxidation by copper or O_2_ take place to afford 3-acyl (2*H*)-indazoles **90** [70].

**Scheme 28:**
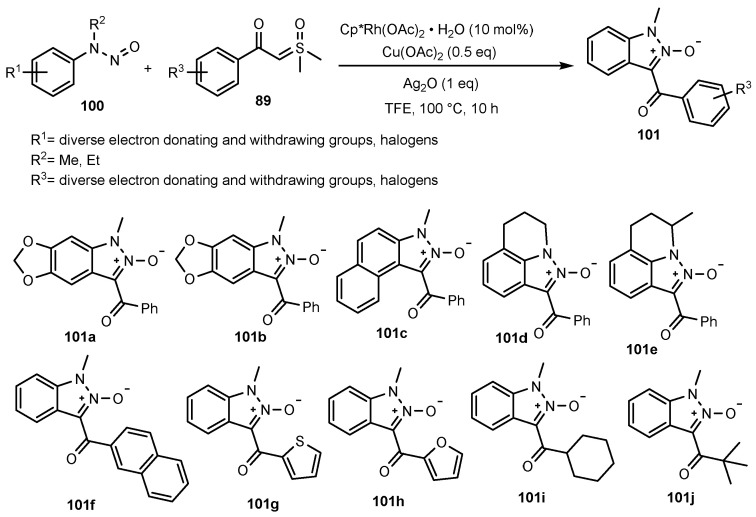
A synthetic route for the preparation of substituted indazole *N*-oxides **101** via the tandem acylmethylation/annulation of *N*-nitrosoanilines **100** with sulfoxonium ylides **89**.

The successful use of *N*-nitrosoanilines **100** in which the *N*-nitroso group plays a dual role as a traceless directing group and an internal nitrosation reagent in the reaction with several sulfoxonium ylides **89** through the Rh(III)/Cu(II)-catalyzed C–H activation and cyclization reaction was demonstrated by Huang et al. in 2020 (Scheme 28). Thus, the subsequent oxidative [4 + 1] annulation reactions of various *N*-nitrosoanilines **100** with sulfoxonium ylides **89** were conducted using Cp*Rh(OAc)_2_ · H_2_O (10 mol%), Cu(OAc)_2_ (0.5 eq), and Ag_2_O (1 eq) in TFE at 100 °C for 10 h. This simple and efficient strategy could be utilized for the synthesis of diverse substituted indazole *N*-oxides **101** with powerful reactivity, good functional-group tolerance, moderate to good yields, and atom- and step-economic reactions under mild conditions. Notably, this unique annulation approach indicates a previously unobserved reactivity model for the *N*-nitroso functional group. The scope and limitations of this cascade acylmethylation/annulation transformation were tested using the reaction of a variety of *N*-nitrosoanilines **100** and sulfoxonium ylides **89**. In general, a diverse array of functional groups (various electron-donating groups and halides) on the *o*-, *m*-, and *p*- positions of *N*-nitrosoaniline rings were possible under these conditions. In the case of *m*-Cl-substituted *N*-nitrosoanilines, a mixture of regioisomers was obtained. *N*-nitrosoanilines bearing two substituents exhibited moderate performances, providing desired products **101a** and **101b**. *N*-alkyl-*N*-nitrosonaphthylamines with different alkyls substituted on the amino group and substituted *N*-nitroso-tetrahydroquinoline were also tolerated, furnishing good to high yields of products **101c–e**. It is noteworthy that sulfoxonium ylides with one or two substitutions on different positions of the aryl ring were very good partners under the standard conditions, achieving moderate to high yields. The naphthyl-derived substrates heterocyclic and alkyl ylides were next examined using this catalytic system, showing moderate yields of **101f–j** [71].

**Scheme 29:**
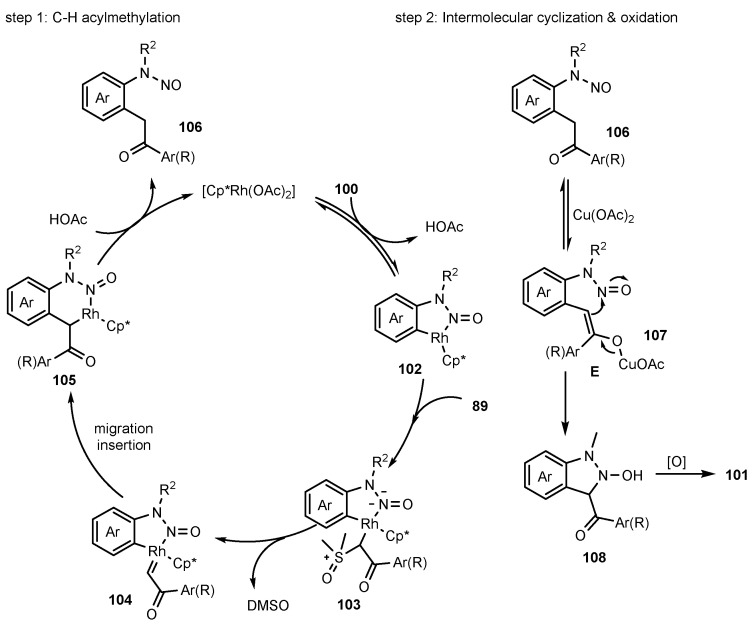
A rational mechanism for the synthesis of substituted indazole *N*-oxides **101** via the tandem acylmethylation/annulation of *N*-nitrosoanilines **100** with sulfoxonium ylides **89**.

A reasonable mechanism is illustrated in Scheme 29. In the first step, coordination/C−H activation with a Rh catalyst affords complex **102**. The insertion of sulfoxonium ylide to the metal center of the previous complex produces a Rh(III) intermediate **103,** followed by releasing a DMSO molecule from **103**. Subsequently, the resulting rhodium *α*-oxo carbene species **104** carry out the migratory insertion of the Rh–C bond to provide a six-membered rhodacyclic intermediate **105**. In the next step, the acylmethylated intermediate **106** is obtained by the protonolysis of the Rh–N and Rh–C bonds. Subsequently, the final product **101** is generated from 106 by Cu(OAc)_2_-mediated intramolecular annulation and oxidation in the presence of either Ag_2_O or Cu(II) additives [71].

**Scheme 30:**
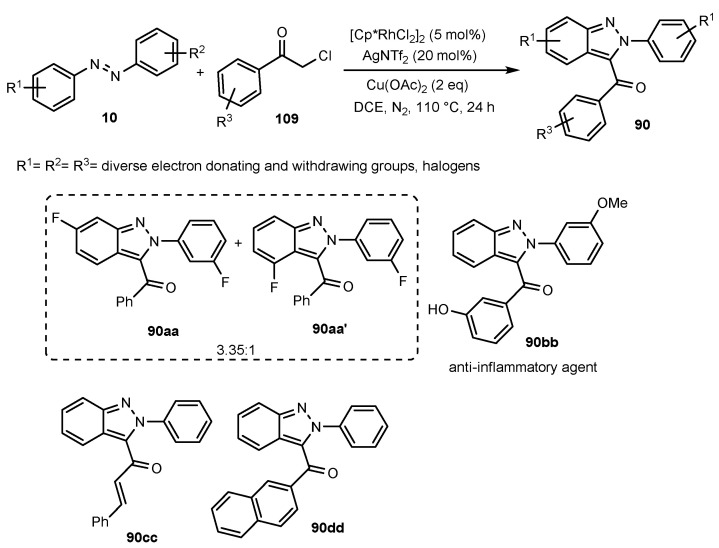
A synthetic route for the preparation of 3-acyl-2-*H*-indazoles **90** via the annulation of azobenzenes **10** with *α*-Cl ketones **109**.

Interesting molecular architectures were achieved by the Rh(III)/Cu(II)-catalyzed [4 + 1] cyclization of azobenzenes **10** with *α*-Cl ketones **109** in excellent yields for more than 30 examples (Scheme 30). The generality and scope of 3-acyl-2*H*-indazoles synthesis by performing the annulation of azobenzenes **10** with *α*-Cl ketones **109** were screened. It was shown that this transformation could tolerate diverse starting materials bearing a variety of functional groups in both azobenzenes **10** and *α*-Cl ketones **109** and provided the desired products **90** in good to excellent yields. Generally, azobenzenes **10** with both electron-donating and -withdrawing functional groups at the *o*, *m,* and *p* positions as well as alkyl-, alkoxy-, or halogen-substituted azobenzenes afforded the desired products in moderate to good yields. It was observed that *m*-F substitution led to a mixture of two regioisomers, **90aa** and **90aa’,** in which the less hindered position was favored. Meanwhile, an unsymmetrical azobenzene-containing methoxy group was annulated with *α*-Cl ketone containing hydroxy at the m position to form an anti-inflammatory agent **90bb** with a high yield in a regioselective manner. Alkyl or heterocyclic azo compounds as substrates have not been applicable in this catalytic system. Various monosubstituted *α*-Cl ketones and disubstituted *α*-Cl ketones were able to undergo this cascade C−H activation/annulation reaction smoothly with azobenzenes to afford the corresponding products in moderate to excellent yields. As a result, substituent effects have no large effect on the yields, although strong electron-withdrawing groups on the *α*-Cl acetophenone aryl ring seem to be more practical for this cascade reaction. Moreover, alkenyl- and naphthyl-substituted scaffolds **90cc–dd** were also successfully used in the reaction to afford the corresponding products in moderate to good yields.

Reactions occurred with high efficiency and with extensive functional-group tolerance under mild reaction conditions. It is worth noting that the efficient production of 3-acyl-2*H*-indazole **90bb** with anti-inflammatory activity in one step ensures the practicability of this approach [72].

**Scheme 31:**
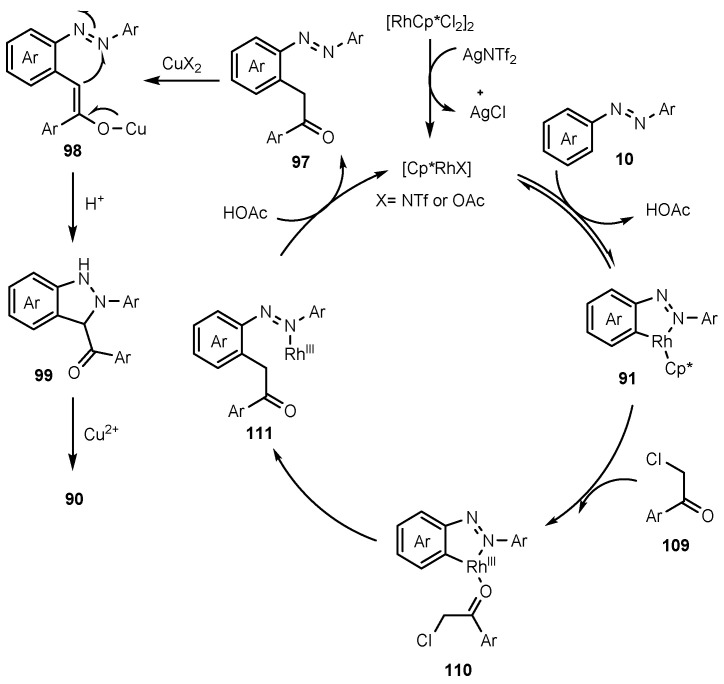
A rational mechanism for the synthesis of 3-acyl-2-*H*-indazoles **90** via the annulation of azobenzenes **10** with *α*-Cl ketones **109**.

A reasonable mechanistic pathway is shown in Scheme 31. The subsequent coordination of azobenzenes **10** with the Rh(III) catalyst and C−H bond activation give a five-membered rhodacyclic intermediate **91**. The *α*-Cl acetophenones **109** coordinate to Rh(III), followed by C-C bond formation. *N*-protonation takes place to produce *α*-aryl-ketone species **97** along with the regeneration of the Rh(III) catalyst. Enol-tautomerization, intramolecular annulation, and oxidation transformations occur in the presence of Cu(II) species to produce target molecules **90** [72].

**Scheme 32:**
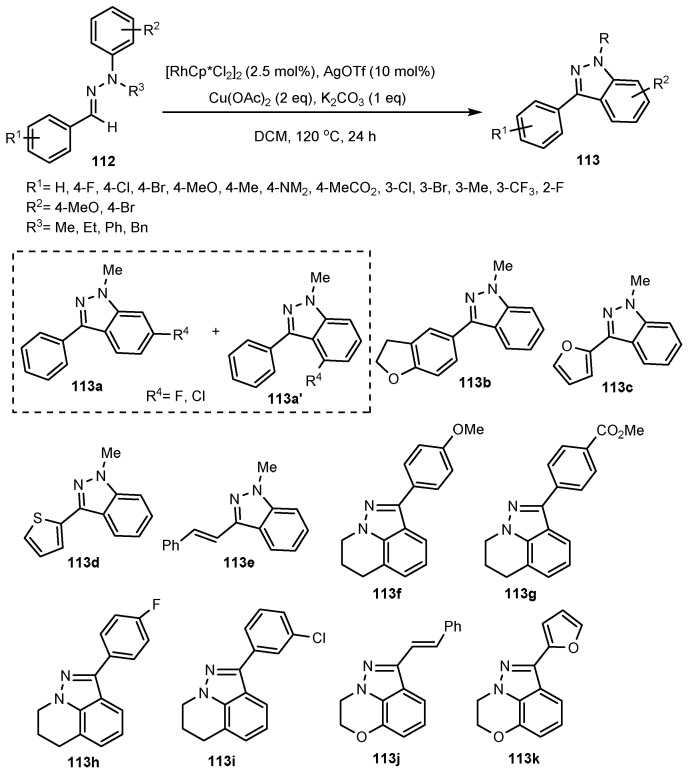
A synthetic route for the preparation of functionalized 1*H*-indazole **113** via the double C−H activation of aldehyde hydrazones **112**.

An efficient and novel C–H activation and C–H/C–H cross-coupling strategy has been developed to realize the synthesis of functionalized 1*H*-indazoles **113** from easily accessible aldehyde phenylhydrazones **112** (Scheme 32). Optimization studies showed that the best yields could be achieved by performing the reaction using a catalytic amount of (RhCp*Cl_2_)_2_/AgOTf, with Cu(OAc)_2_ as the oxidant and K_2_CO_3_ as the base at 120 °C in 1,2-dichloroethane. Initially, the scope of the substrates with respect to different *N*-alkyl (Me, Et, and Bn) or aryl groups was tested. It was observed that these functional groups had only a moderate effect on the reaction, generating the desired compounds in very good to high yields. Whereas benzaldehyde derivatives **112** containing different electron-rich and electron-poor functional groups on the para- or meta-positions of the aryl ring yielded good to high yields of the corresponding indazoles, ortho-substituted benzaldehyde derivatives displayed less efficacity in this reaction, delivering the corresponding indazole **113** in a low yield. Heteroaromatics such as furan- and thiophene-aldehyde starting materials were also well-tolerated under this catalytic strategy, leading to the expected indazoles in acceptable yields. In the case of substrates substituted at the meta-position of the *N*-aryl ring, the regioselectivity was moderate, showing a mixture of products **113a** and **113a’** that had reacted at both positions. Some complex products **113b–k** synthesized by this methodology are illustrated in Scheme 32. In order to highlight the importance of this strategy, the authors extended the procedure to an easy preparation of certain important and bioactive products. These significant products displayed good 5-HT4/5-HT3 receptor antagonist activity. This transition metal strategy reveals good scalability, good functional-group compatibility, wide substrate scope, and moderate to high yields under mild reaction conditions. The combination of mechanistic experiments and DFT calculations indicates C(aryl)-H bond activation followed by a C(aldehyde)–H bond activation, and the reductive elimination process occurs to form a variety of targeted products **113** [73].

### 2.10. Synthesis of Indazoles Using Rhodium, Silver, Copper, and Zinc Salts

**Scheme 33:**
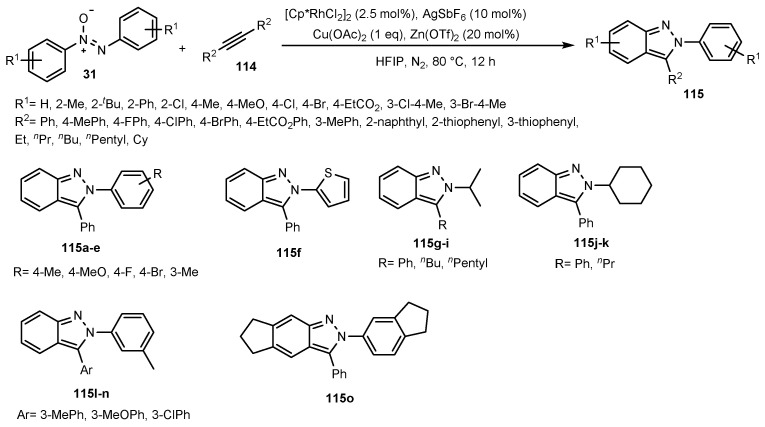
A synthetic route for the preparation of 2*H*-indazole derivatives **115** via the [4 + 1] annulation of azoxy compounds **31** with alkynes **114**.

Efficient rhodium(III)-catalyzed regioselective C–H activation/cyclization of an azoxy compound **31** with an alkyne **114** as the coupling partner has been realized via a [4 + 1] cycloaddition rather than a normal [4 + 2] mode (Scheme 33). The processes of cyclative capture, oxygen-atom transfer, and C≡C triple bond cleavage are the key steps of this reaction. The coupling of azoxy compounds **31** with alkynes **114** was conducted using [Cp*RhCl_2_]_2_ (2.5 mol%) as a precatalyst, AgSbF_6_ (10 mol%), Cu(OAc)_2_ (1.0 equiv), Zn(OTf)_2_ (20 mol%), and HFIP (1.0 mL) under N_2_ at 80 °C for 12 h. It seems AgSbF_6_ and Cu(OAc)_2_ are exploited to activate the Ru catalyst before or during the catalytic cycle. The authors mentioned no exact role for the Lewis acid Zn(OTf)_2_. Initially, the generality of this catalytic system was studied with respect to the azoxy substrates **31**. The reactions with symmetrical azoxybenzenes containing electron-rich substituents including Me, *t*-Bu, and OMe as well as electron-withdrawing substituents such as F, Cl, Br, and COOEt proceeded smoothly to give indazoles **115** in moderate to excellent yields. It is worth noting that a variety of meta-substituted azoxybenzenes were used in the reaction to produce the corresponding products **115** in good to high yields while reacting at the less sterically hindered position. The strategy could also be extended to unsymmetrically substituted diaryldiazene oxide substrates **31** (leading to **115a–n**). Even the monoaryldiazene oxide substrates were well-tolerated and successfully reacted with diphenylacetylene to produce the expected indazoles **115g–k** in satisfactory yields. While the reaction was compatible with a wide range of azoxybenzenes, substrates functionalized by substituents such as Me, OMe, Cl, and Br at the ortho-position of the NO-phenyl ring failed to give corresponding products, probably due to steric congestion. Furthermore, the scope and limitations of the reaction were tested by the exploitation of alkyne substrates. As summarized in Scheme 33, different symmetrical diarylalkynes with both electron-rich and electron-poor substituents were successfully utilized under the optimized reaction conditions, which gave indazoles **115** in moderate to good yields. It is noteworthy that this catalytic system showed complete compatibility with diarylacetylene substrates bearing 2-naphthyl, 2-thienyl, or 3-pyridyl scaffolds as well as dialkylalkyne substrates. Bis(trimethylsilyl)acetylene or monosubstituted alkyne substrates failed in this [4 + 1] annulation reaction. This strategy demonstrates various outstanding features, such as broad substrate scope, good functional-group tolerance, and operational convenience, which enable regioselective access to different 2,3-disubstituted 2*H*-indazoles **115** in moderate to high yields [74].

### 2.11. Synthesis of Indazoles Using Cobalt and Copper Salts

**Scheme 34:**
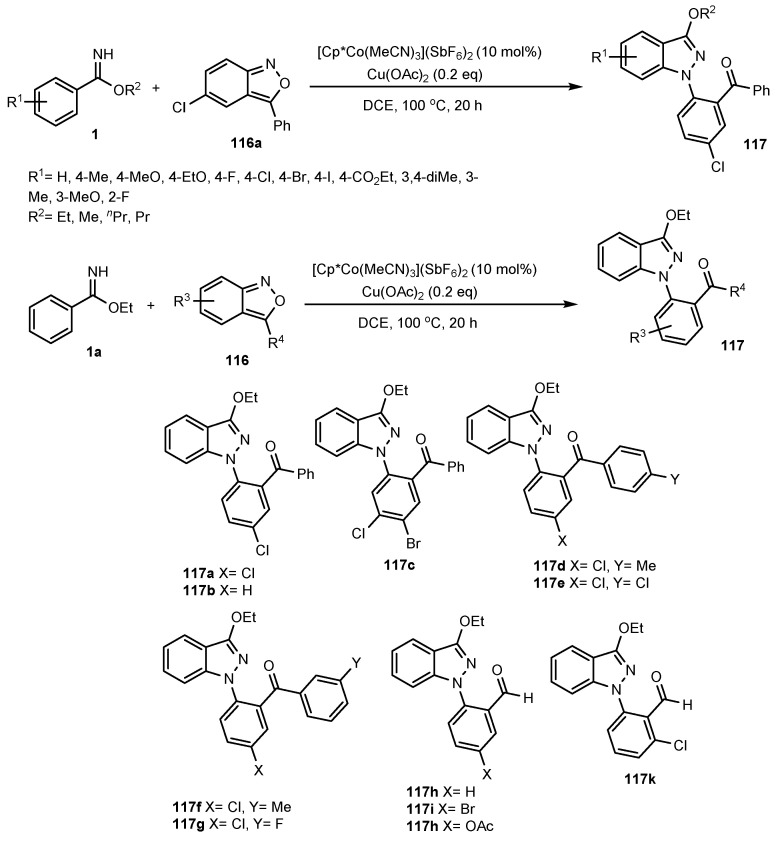
A synthetic route for the preparation of *H*-indazoles **117** via the C−N/N−N coupling of imidates **1** with anthranils **116**.

A Co/Cu-catalyzed C–H activation/oxidative coupling of imidate esters **1** with anthranils **116** in DCE at 100 °C (sealed tube) under N_2_ atmosphere within 20 h for the synthesis of 1*H*-indazole derivatives **117** was described by Li et al. (Scheme 34). In this paper, anthranils **116** were exploited as novel bifunctional aminating reagents and organic oxidants under Co/Cu catalysis, affording a broad range of 1*H*-indazoles **117** in high yields (up to 99% yield) with excellent selectivity. It seems that the formation of the N–N bond involves Cu catalysis.

Initially, anthranil **116a** was reacted with a range of imidates **1** containing both electron-donating and -withdrawing functional groups at the para-site of the phenyl ring. The results summarized in Scheme 34 demonstrate that this methodology is useful for the coupling of **116a** with a range of functionalized imidates **1**. The meta-substituted imidates were examined for the synthesis of the product **117** under standard conditions, with the C–N/N–N coupling of imidates **1** with anthranil **116a** taking place at the less hindered position. In the next step, the substrate scope of anthranil derivatives **116** under standard reaction conditions was investigated. Various substituents, including halogens and phenyl on different positions of the anthranil ring and a phenyl group substituted into the 3-position of the anthranil ring, were all applicable, leading to the desired indazole derivatives in satisfactory yields. Although nonsubstituted anthranil was an effective substrate, the reaction rate was decreased. In this case, the utilization of pivalic acid additive facilitated the C−H activation transformation. When the reaction was carried out with 2-azidobenzaldehyde precursors instead of anthranils, poor results were obtained [75].

**Scheme 35:**
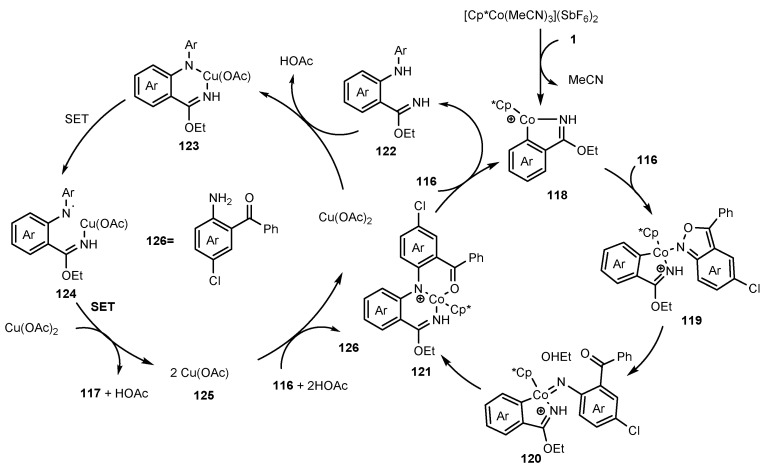
A rational mechanism for the synthesis of *H*-indazoles **117** via the C−N/N−N coupling of imidates **1** with anthranils **116**.

A plausible reaction mechanism for the Co(III)/Cu(II)-catalyzed synthesis of 1*H*-indazoles **117** from the coupling of imidates **1** with anthranils **116** is shown in Scheme 35. The mechanism comprises the cyclometalation of the imidate **1**, the coordination of anthranil to intermediate **118**, intramolecular N−O bond cleavage forming **120**, the migratory insertion of the Co−aryl bond of **122** into the nitrene, and the coordination of an imidate to **121,** along with releasing the aminated intermediate **122**. In a successive catalytic cycle, the coordination of Cu(OAc)_2_ to **122** and a double single-electron transfer occur to form target indazole **117** [75].

### 2.12. Synthesis of Indazoles Using Palladium Salts

**Scheme 36:**
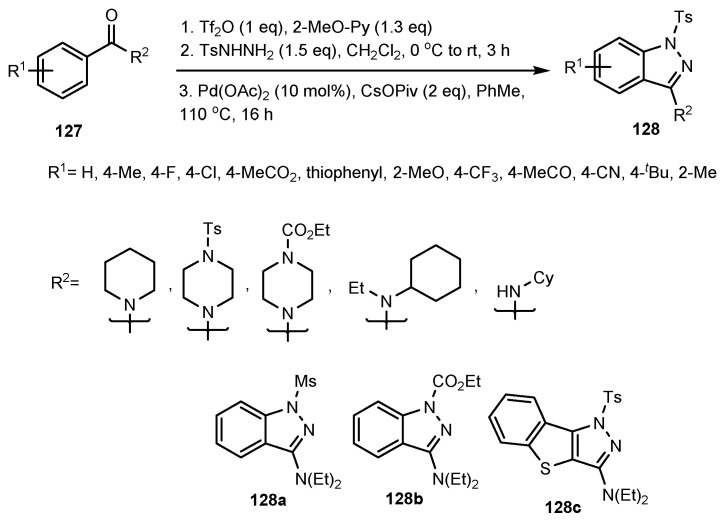
A synthetic route for the preparation of 3-aminoindazoles **128** from tertiary amides **127**.

The Pd-catalyzed C−H activation/annulation of substrates **127** was achieved by Charette in 2015. A series of 3-aminoindazoles **128** was produced with moderate to high yields (Scheme 36). In this reaction, the intermediate amidrazones were initially synthesized using an easy route from amide substrates **129** via (1) triflic anhydride activation and (2) condensation with hydrazines, to then (3) undergo a Pd-catalyzed C−H amination reaction. Structurally diverse amidrazones bearing various functional groups on the arene or nitrogen substituents revealed acceptable reactivity, yielding the expected indazoles **128**. As illustrated in Scheme 36, starting substrates with several cyclic and acyclic amine scaffolds can be applicable in the reaction. Various types of para-substituted substrates were investigated, delivering the desired indazoles **128** in yields of 15%−70%. In the case of a meta-substituted substrate (**127** substituted with OMe at the meta-position), a 4:1 mixture of regioisomers was obtained. Unfortunately, ortho-substituted substrates did not work in the reaction. Notably, the reaction was also effective using a substrate containing a 2-benzothienyl scaffold, achieving 3-aminoindazole fused heterocycle **128c**. The author offered no mechanistic explanation, but this may likely involve classical Pd-catalyzed C−H activation followed by cyclization. The current strategy offers an efficient procedure for the synthesis of 3-aminoindazoles **128**, which are easily synthesized from amide substrates in two steps [24].

### 2.13. Synthesis of Indazoles Using Palladium Salt in the Presence of Cobalt Salts

**Scheme 37:**
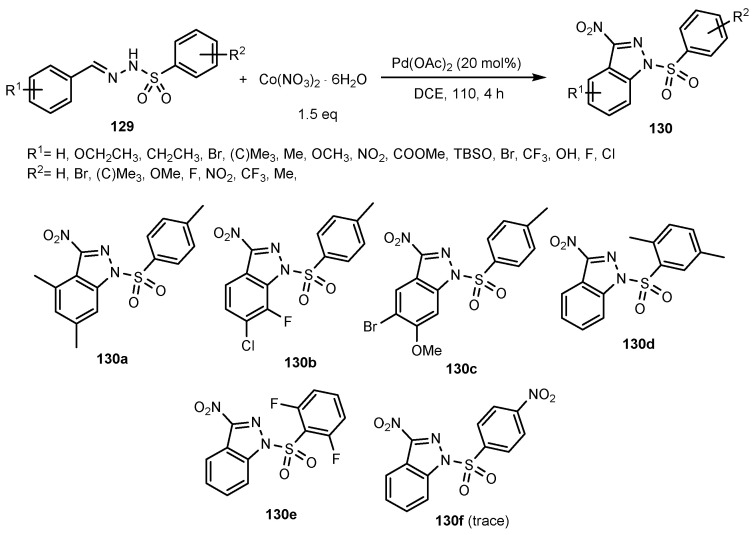
A synthetic route for the preparation of 3-nitro-1-(phenylsulfonyl)-1*H*-indazole derivatives **130** via the direct C−H nitration and intramolecular C−H functionalization of **129**.

An efficient Pd-catalyzed C–H functionalization/nitration/cyclization sequence for the synthesis of 3-nitro-1-(phenylsulfonyl)-1*H*-indazole derivatives **130** under mild reaction conditions was reported for the first time by Xia et al. (Scheme 37). The chelate-assisted cleavage of two C–H bonds is the key step in this transformation. The reaction was conducted using substrates **129** in the presence of 20 mol% Pd(OAc)_2_ and 1.5 eq Co(NO_3_)_2_ · 6H_2_O in DCE in a sealed tube at 110 °C for 4 h. Co(NO_3_)_2_ · 6H_2_O has been used as an efficient nitration agent. A variety of functional groups on the para- and meta-positions of the benzylidene part were well-tolerated with little electronic dependence, providing, in most cases, 3-nitro-1-(phenylsulfonyl)-1*H*-indazoles **130** in excellent yields with high selectivity. The substrates substituted at the ortho-positions of the benzylidene scaffold underwent the C−H nitration and intramolecular C−H activation sequence uneventfully to give the target indazoles in more than 80% yields. As shown in Scheme 37, disubstituted substrates could also be successfully employed to deliver the indazoles **130a–e**. However, the di-MeO substrate failed to provide the desired product under the optimized reaction conditions. It was also found that various substrates containing substitutions at different positions of the phenylsulfonyl group were all tolerated to provide the desired 3-nitro-1-(phenylsulfonyl)-1*H*-indazole derivatives **130** with high yields for most cases. In the case of 4-NO_2_, the reaction did not work under optimized reaction conditions (only a trace amount of target product **130f** was produced). In general, this transformation proceeded smoothly and could tolerate various substituents, providing easy access to a series of new indazoles [76].

**Scheme 38:**
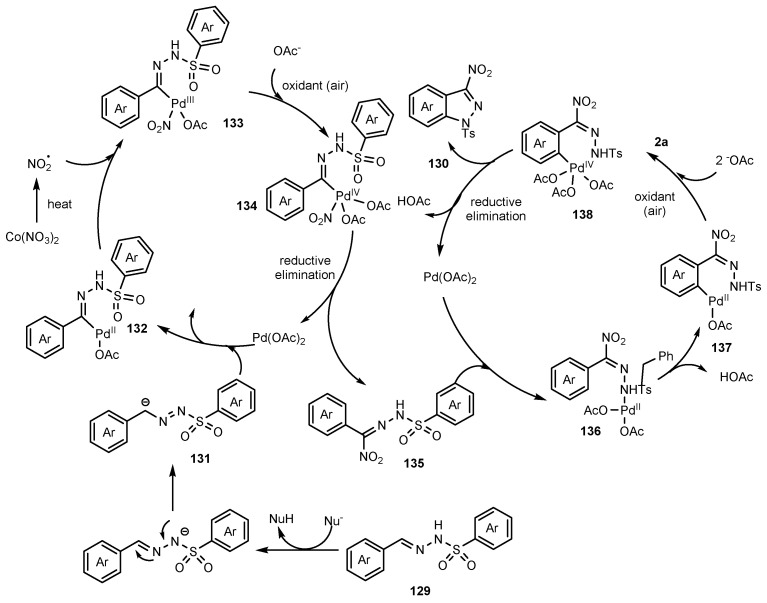
A rational mechanism for the synthesis of 3-nitro-1-(phenylsulfonyl)-1*H*-indazole derivatives **130** via the direct C−H nitration and intramolecular C−H functionalization of **129**.

A plausible mechanism for the synthesis of **130** is outlined in Scheme 38. The mechanism initially comprises the complexation of Pd(OAc)_2_ with the anionic intermediate **131**, the combination of in situ-generated NO_2_^•^ with intermediate **132**, and oxidation and reductive elimination to form intermediate **135** and Pd(OAc)_2_. Next, the coordination of intermediate **135** with Pd(OAc)_2_ followed by C−H bond activation affords six-membered Pd(II) species **137**. Finally, oxidation to a Pd(IV) species **138** by air and reductive elimination take place to produce corresponding product **130** and the Pd(OAc)_2_ [76].

### 2.14. Synthesis of Indazoles Using Palladium, Cerium, and Iron Salts

**Scheme 39:**
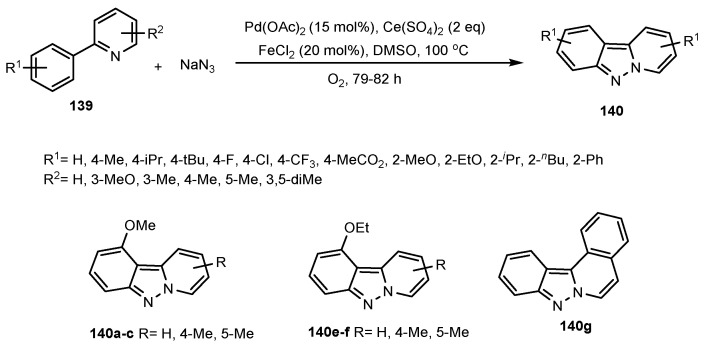
A synthetic route for the preparation of pyrido[1,2-*b*]indazoles **140** via the tandem C−H azidation and N-N bond formation of arylpyridines **139**.

A novel, efficient, rapid, and versatile protocol to generate pyrido[1,2-*b*]indazole derivatives **140** through the C–H activation/azidation of arylpyridines **139** has been developed by Jiao et al. (Scheme 39). The synthetic approach involves C−H azidation and N-N bond formation, which was carried out at 100 °C using 15 mol% Pd(OAc)_2_, 20 mol% FeCl_2_, and Ce(SO_4_)_2_ in DMSO at 100 °C under O_2_ atmosphere for 79–82 h. The scope of this reaction was first studied using the 3-methoxy-2-arylpyridine substrate and its derivatives. The substituent effect (electron-rich or electron-poor substituents) on the 3-methoxy-2-arylpyridine substrate was checked, showing a moderate effect on the yields. The 3-methyl-2-phenylpyridine substrate was explored as a further substrate, affording a satisfactory yield of the desired product. 4-Methyl, 5-methyl, or 3,5-dimethyl-substituted-2-phenylpyridines could also be successfully employed to provide the desired pyrido[1,2-*b*]indazoles in acceptable yields. It is worth noting that sterically hindered substrates could also be utilized, only providing the corresponding pyrido[1,2-*b*]indazoles **140a–f** in moderate yields. As expected, substrates with either electron-rich substituents such as Me and *t*-Bu or electron-poor substituents such as F and Cl at the para-position of the phenyl ring also proved to be suitable for this Pd-catalyzed tandem C−H azidation and N-N bond formation. Finally, 1-phenylisoquinoline yielded the corresponding product **140g** under optimized reaction conditions in a good yield. Involving direct C–N (via 2-pyridyl-directed Pd-catalyzed azidation) and N–N (via concerted nitrogen loss/ring closure) formations, this procedure proceeds under mild conditions, thus demonstrating a methodology for the synthesis of [*b*]fused indazoles in a single step [77].

### 2.15. Synthesis of Indazoles Using Palladium, Copper, and Silver Salts

**Scheme 40:**
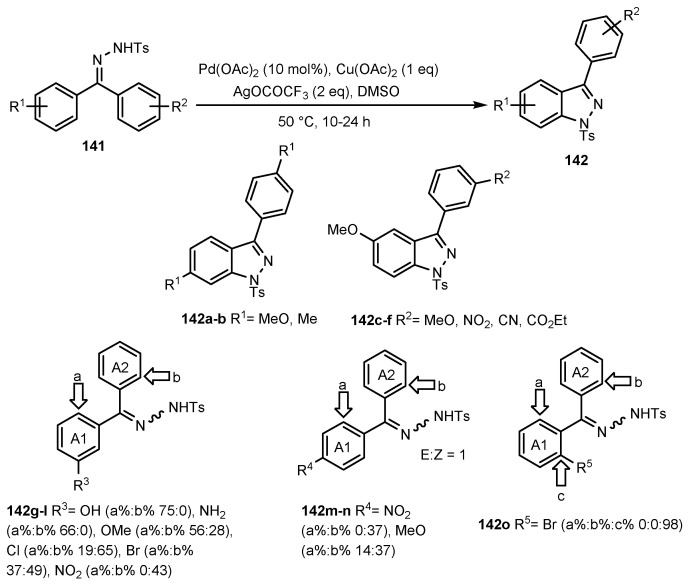
A synthetic route for the preparation of 3-aryl/alkylindazoles **142** via the C−H activation/intramolecular amination reaction.

An efficient procedure for the mild synthesis of 3-aryl/alkylindazoles **142** catalyzed by palladium via a direct C−H activation/intramolecular amination sequence of hydrazone substrates **141** was presented by Hiroya (Scheme 40). This catalytic C−H activation/intramolecular amination reaction was performed involving 10 mol% Pd(OAc)_2_, 1 equiv Cu(OAc)_2_, and 2 equiv AgOCOCF_3_ in DMSO at 50 °C for 10–24 h using hydrazone substrates **141**. This reaction was investigated using different substrates containing both electron-donating and -withdrawing functional groups on the benzene rings. As illustrated in Scheme 40, the transformation with two para-methoxy substituents gave only a 13% yield of product **142a**. In contrast, the compound containing two para-methyl substituents provided the expected product **142b** with a 73% yield. Surprisingly, the compound containing two meta-methoxy substituents proceeded successfully to afford only one product in a regioselective manner. Next, the starting materials containing two different groups at the meta-position reacted only at the 6-position on the more electron-donating aromatic ring, evidencing that both steric and electronic factors affect the outcome (see the products **142d–f**). The products **142** were achieved in acceptable yields using diverse monosubstituted substrates. As expected, the reaction pathway was completely dependent on the nature of the functional groups on the benzene rings. The electron-rich donating substituents mainly oriented the reaction towards the A1 arene **142g–h**, whereas hydrazones containing an electron-poor substituent on the arene produced the corresponding indazoles on the nonsubstituted A2 arene **142d–f**. Notably, a C−H activation/intramolecular amination sequence took place preferentially on the A2 arene when para-substituted hydrazones containing electron-rich or -poor substituents were explored **142m–n** [78].

### 2.16. Synthesis of Indazoles Using Cobalt Salts

**Scheme 41:**
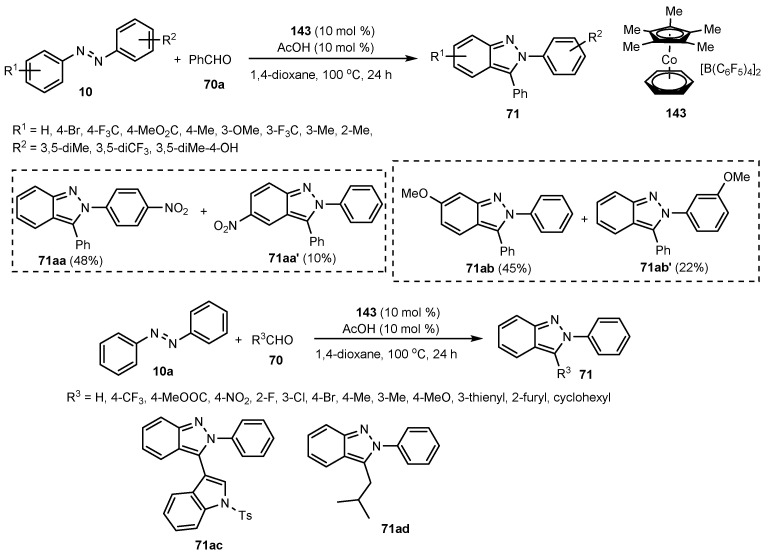
A synthetic route for the preparation of indazoles **71** via C–H bond functionalization/addition/cyclization cascades.

A novel efficient synthesis of indazoles **71** via sequential C–H bond functionalization/addition/cyclization was reported by Ellman (Scheme 41). The target products **71** were synthesized via a convergent one-step route using a novel air-stable cationic Co(III) catalyst. The reaction has been previously performed using 5 mol% (Cp*RhCl_2_)_2_ and 20 mol% AgSbF_6_ in dioxane at 80 °C for 24 h. Although this system is efficient for the synthesis of indazoles, this chemistry displays some disadvantages, especially using an expensive Rh catalyst as well as the availability of Rh in the nature [66]. The reaction was carried out in the presence of 10 mol% co-catalyst **143** and 10 mol% AcOH in 1,4-dioxane (2.0 M) at 100 °C for 24 h and afforded the desired products **71** mostly with acceptable yields. First, the substrate scope of azobenzenes **10** was explored. Here, several symmetric and unsymmetrical azobenzenes containing electron-rich and electron-poor substituents were all well-tolerated and delivered the desired compounds in a regioselective manner. However, in the case of the para-nitro or meta-methoxy substituted azobenzene, a mixture of products **71aa**, **71aa’**, **71ab,** and **71ab’** was obtained. Different 3,5-disubstituted azobenzene substrates bearing a variety of substituents with donating or deficient natures were proven to afford satisfactory yields involving the less sterically hindered ring in the cyclization. Moreover, aromatic aldehydes containing electron-rich and electron-poor substituents as well as heteroaromatic aldehydes successfully reacted with azobenzene to form the corresponding indazoles in acceptable yields. The results of this study are illustrated in Scheme 41 [79].

### 2.17. Synthesis of Indazoles Using Rhenium Salt in the Presence of Sodium Acetate

**Scheme 42:**
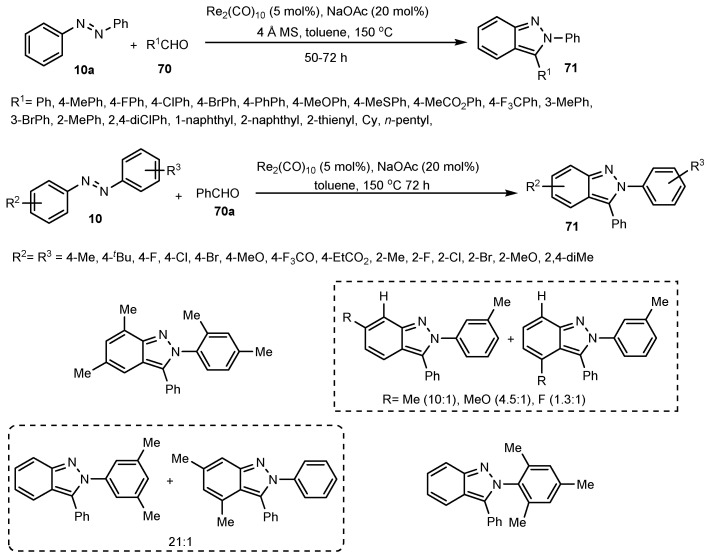
A synthetic route for the preparation of indazole derivatives **71** via the [4 + 1] annulation of azobenzenes **10** with aldehydes **70**.

Wang and co-workers employed the first Re catalysis for the [4 + 1] annulation of azobenzenes **10** with aldehydes **70** to furnish 2*H*-indazoles **71** using catalytic amounts of Re_2_(CO)_10_ and NaOAc in toluene at 150 °C within 50–72 h in an oven-dried Schlenk tube (Scheme 42). In comparison to similar works performed by Rh [66] and Co [79] catalysts, Re is less expensive than Rh but more expensive than a Co catalyst. It should be noted that Co and Rh catalysts could provide milder conditions for the reaction. The acetate could potentially enable an acceleration effect in the Re-catalyzed C–H activation of azobenzenes **10**. Initially, the scope of aldehyde substrates **70** was tested with both electron-rich and -poor functional groups on the aromatic ring, producing the target products in moderate to high yields. A wide variety of aldehydes **70** with substituents such as F, Cl, Br, MeS, CO_2_Me, and CF_3_ also coupled with different azobenzenes **10** to give corresponding products **71** in satisfactory yields. The reaction progressed smoothly using naphthaldehydes and thiophene-2-carbaldehyde to afford indazole derivatives **71** in good yields. When aliphatic aldehydes were introduced into this reaction, the yield was decreased. The scope and limitations of the reaction were screened by focusing on azobenzene derivatives **10**. The reaction proceeded smoothly with a variety of para-substituted azobenzenes, giving the corresponding indazoles **71** in good to high yields. Moreover, a variety of substituents on the ortho-position of azobenzenes were well-tolerated under standard conditions. In the case of disubstituted azobenzene (two methyl groups at the 2- and 4-positions of both rings), the reaction efficiently underwent the C−H functionalization/[4 + 1] annulation and generated the expected product in a high yield. Meanwhile, meta-methyl azobenzene produced a moderate yield of the corresponding product with high regioselectivity. The structures of some target products are shown in Scheme 42. No significant effect on the yield was observed when azobenzene was replaced with a bulky (mesityl) analog [80].

**Scheme 43:**
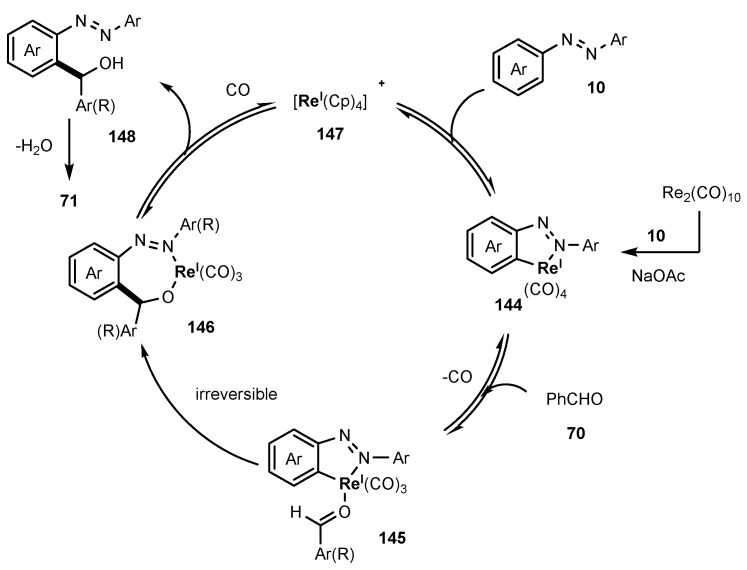
A rational mechanism for the synthesis of indazole derivatives **71** via the [4 + 1] annulation of azobenzenes **10** with aldehydes **70**.

A reasonable reaction mechanism for the rhenium-catalyzed [4 + 1] annulation of azobenzenes **10** with aldehydes **70** is shown in Scheme 43. First, C−H is activated in the presence of Re_2_(CO)_10_/NaOAc to afford a cyclic Re(I)-complex **144**. In the next step, complex **145** bearing aldehyde is generated via an exchange between a CO ligand and an aldehyde. Finally, an irreversible aldehyde insertion to the Re−aryl bond, protonation, and an intramolecular nucleophilic substitution transformation followed by rearomatization produce the final product **71** [80].

### 2.18. Synthesis of Indazoles Using Copper Salts

**Scheme 44:**
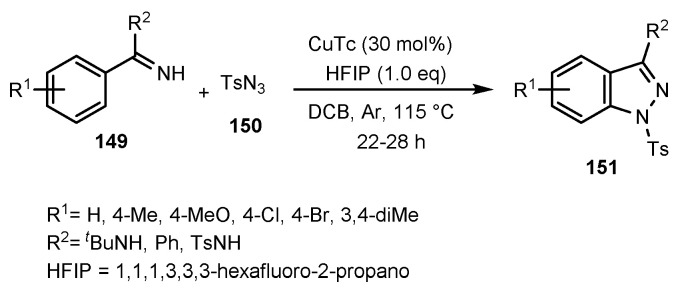
A synthetic route for the preparation of indazole derivatives **151** via C(sp^2^)−H amidation **149** with azides **150** as amino sources.

A highly regioselective synthesis of indazoles **151** was reported by Glorius via a copper-catalyzed C−H amidation process transformation. In this reaction, azides **150** were used as amino sources, and no oxidizing agent was used. The reaction exploited amidines or imine **149** to perform a tandem C−N/N−N bond-forming transformation for the production of indazole derivatives **151** in one pot (Scheme 44). All annulation reactions were carried out in 1,2-dichlorobenzene (DCB) at 115 °C using copper(I) thiophene-2-carboxylate (CuTc) (30 mol%) and 1,1,1,3,3,3-hexafluoro-2-propanol (HFIP) (1.0 eq) under Ar in a sealed tube. The *N*-tert-butylarylamidine substrates **149** bearing Me, MeO, Cl, or Br have been successfully employed in the Cu-catalyzed synthesis of *N*-tert-butyl-3-aminoindazoles using coupling with TsN_3_ via a tandem C−N/N−N bond-forming reaction. A more detailed explanation of the mechanism includes a Cu-metallacycle, then nitrene coordination, migratory insertion, and reductive amination. The reaction of both tosyl-protected and free benzimidamide failed to form the corresponding products under optimized conditions. In addition, diphenylmethanimine as the starting material was examined, showing the expected indazole formation [81].

**Scheme 45:**
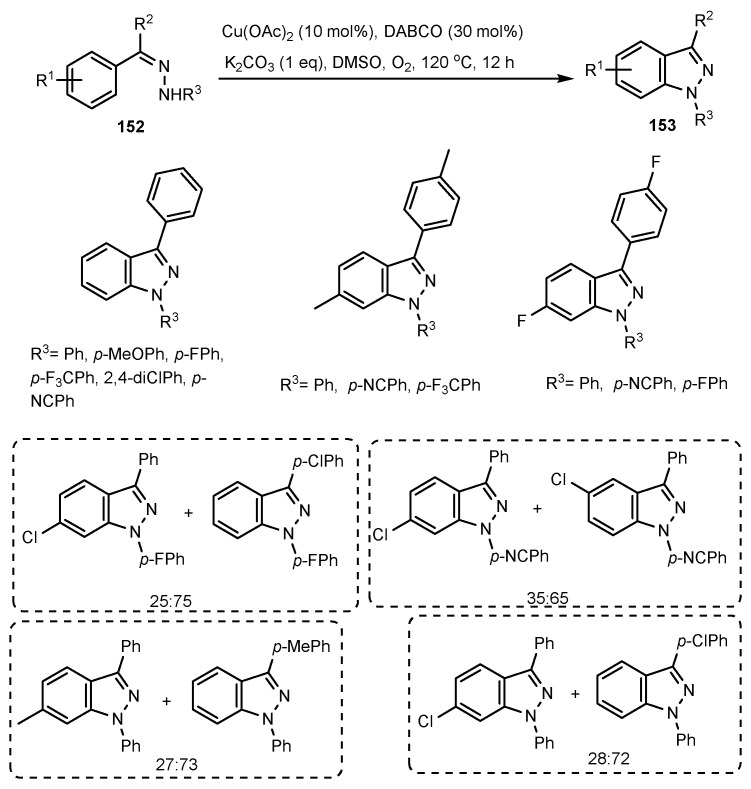
A synthetic route for the preparation of indazole derivatives **153** via aerobic C(sp^2^)−H functionalization/C−N bond formation.

An atom-economical protocol for the practical preparation of indazoles **153** through the Cu-catalyzed direct aerobic oxidative C(sp^2^)–H amination of benzophenone hydrazones **152** has been illustrated by Jiang et al. (Scheme 45). The synthesis of indazoles **153** was carried out successfully through an aerobic C(sp^2^)−H activation for C−N bond formation in the presence of catalytic amounts of Cu(OAc)_2_ and DABCO in DMSO within 12 h in 70–86% yields. The substrate scope of the reaction was then tested. As shown in Scheme 45, the reactions of substrates **152** with substituents such as F, Cl, CF_3_, and CN were then explored. These cascade reactions proceeded smoothly and afforded the corresponding indazoles **153** in moderate to good yields under standard conditions. Notably, substrates **152** containing an electron-rich substituent produced the desired indazoles in relatively higher yields than those with electron-poor substituents. It is worth noting that by using benzophenone hydrazone **152** containing two electronically unsymmetrical aromatic groups, a mixture of isomers was obtained, indicating that the reaction does not proceed in a regioselective manner. Some complex products synthesized by this Cu-catalyzed direct aerobic oxidative C(sp^2^)–H amination are illustrated in Scheme 45. The procedure has the privilege of good to high yields and tolerates a variety of substituents.

**Scheme 46:**
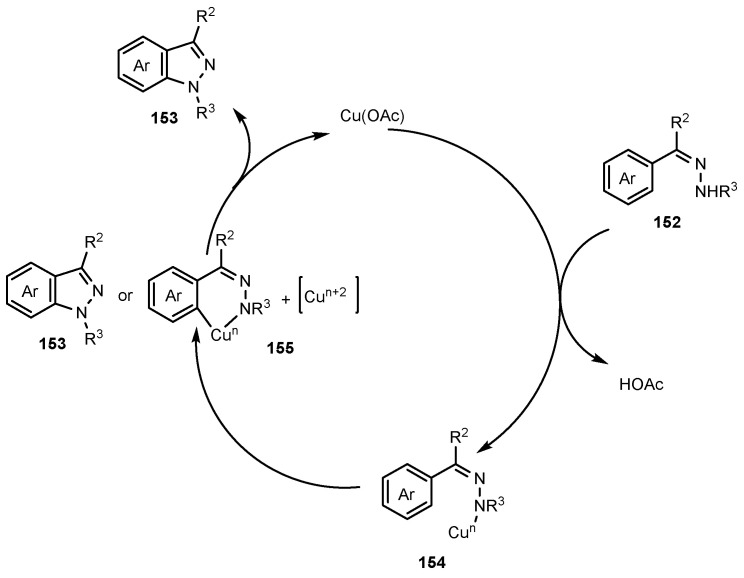
A rational mechanism for the synthesis of indazole derivatives **153** via aerobic C(sp^2^)−H functionalization/C−N bond formation.

First, the Cu(OAc)_2_ is probably coordinated with substrate **152,** resulting in a Cu−N adduct **154**. Subsequently, the C−H activation and C-N bond-forming transformation take place to afford the target molecules **153** (Scheme 46) [23].

## 3. Conclusions

The *N*-heterocycles show a wide structural variety that is beneficial for the investigation of further therapeutic agents for enhancing the pharmacokinetics and other physicochemical properties. Indazole derivatives are a significant category of *N*-heterocyclic five-membered systems that display a broad range of applications in biology, chemistry, and materials science. The investigation of indazole derivatives has become a rapidly evolving and increasingly active subject in therapeutic science. Meanwhile, transition-metal-catalyzed C–H activation/annulation strategies for the one-pot synthesis of indazoles has been growing steadily in synthetic organic chemistry. They are a superior class of transformations that serve as powerful tools to access a wide range of indazoles. Numerous outstanding results have been reported for the synthesis of these structurally diverse indazole frames via transition-metal-catalyzed C–H activation/annulation reactions. The results of these articles have not previously been thoroughly studied or reviewed. This review article presents several atom-economical strategies for synthesizing indazole derivatives from accessible substrates through metal-catalyzed C−H functionalization and annulation transformations. The mentioned reactions feature easily available substrates, relatively good to high yields, large functional-group tolerance, diversity, and a high complexity of products under mild reaction conditions, furnishing practically useful methods to access structurally diverse indazoles.

## Data Availability

The data presented in this study are available on request from the corresponding author.
